# Comparative Analysis of CRISPR/Cas9 Delivery Methods in Marine Teleost Cell Lines

**DOI:** 10.3390/ijms262110703

**Published:** 2025-11-03

**Authors:** Álvaro J. Arana, Sara Veiga-Rua, Diego Cora, Manuel A. Gónzalez-Gómez, Ana Seijas, Maialen Carballeda, David Polo, Alberto Cuesta, Yolanda Piñeiro, José Rivas, Mercedes Novo, Wajih Al-Soufi, Paulino Martínez, Laura Sánchez, Diego Robledo

**Affiliations:** 1 Departamento de Zooloxía, Xenética e Antropoloxía Física, Facultade de Veterinaria, Universidade de Santiago de Compostela, 27002 Lugo, Spain; sara.veiga.rua@rai.usc.es (S.V.-R.);; 2 Departamento de Química Física, Facultade de Ciencias, Campus Terra, Universidade de Santiago de Compostela, 27002 Lugo, Spain; diego.cora.calvo@usc.es (D.C.);; 3 NANOMAG Laboratory, Department of Applied Physics, Materials Institute (iMATUS), Universidade de Santiago de Compostela, 15782 Santiago de Compostela, Spain; 4 NanoToxGen Group, CICA—Centro Interdisciplinar de Química e Bioloxía, Universidade da Coruña, Rúa As Carballeiras, 15071 A Coruña, Spain; 5 Departamento de Biología, Facultade de Ciencias, Universidade da Coruña, Campus da Zapateira, 15071 A Coruña, Spain; 6 Fish Innate Immune System Group, Department of Cell Biology, Faculty of Biology, University of Murcia, 30100 Murcia, Spain; 7 The Roslin Institute and Royal (Dick) School of Veterinary Studies, University of Edinburgh, Easter Bush Campus, Edinburgh EH25 9RG, UK

**Keywords:** CRISPR, Cas9, gene editing, editing efficiency, aquaculture, sea bass, sea bream

## Abstract

Gene editing technologies such as CRISPR/Cas9 have revolutionized functional genomics, yet their application in marine fish cell lines remains limited by inefficient delivery. This study compares three delivery strategies—electroporation, lipid nanoparticles (LNPs), and magnetofection using gelatin-coated superparamagnetic iron oxide nanoparticles (SPIONs)—for CRISPR/Cas9-mediated editing of the *ifi27l2a* gene in DLB-1 and SaB-1 cell lines. We evaluated transfection and editing efficiency, intracellular Cas9 localization, and genomic stability of the target locus. Electroporation achieved up to 95% editing in SaB-1 under optimized conditions, but only 30% in DLB-1, which exhibited locus-specific genomic rearrangements. Diversa LNPs enabled intracellular delivery and moderate editing (~25%) in DLB-1 but yielded only minimal editing in SaB-1, while SPION-based magnetofection resulted in efficient uptake but no detectable editing, highlighting post-entry barriers. Confocal imaging and fluorescence correlation spectroscopy suggested that nuclear localization and Cas9 aggregation may influence editing success, highlighting the importance of intracellular trafficking in CRISPR/Cas9 delivery. Our findings demonstrate that CRISPR/Cas9 delivery efficiency is cell line-dependent and governed by intracellular trafficking and genomic integrity. These insights provide a practical framework for optimizing gene editing in marine teleosts, advancing both basic research and selective breeding in aquaculture.

## 1. Introduction

Gene editing using CRISPR/Cas9 is radically transforming aquaculture biotechnology by enabling the precise modification of genes associated with economically relevant traits such as growth, sterility, and disease resistance [1,2,3]. This tool has been applied in fish embryos via microinjection, achieving high knockout rates in species such as zebrafish, Atlantic salmon, and cod, but its use in cell lines is limited by several species-specific barriers that complicate the direct implementation of protocols developed for mammalian systems. These barriers include low transfection efficiency, slow cell proliferation, differences in promoter compatibility, and reduced nuclear access of large plasmids [4,5,6,7,8,9].

One of the main factors underlying efficient genome editing in fish cell lines is the choice of delivery system, which must overcome unique physiological and molecular barriers associated with teleost species [10]. Electroporation of Cas9–sgRNA ribonucleoprotein (RNP) complexes has emerged as a highly effective strategy, with editing efficiencies of up to 61.5% in medaka and 39% in rainbow trout, while avoiding genomic integration [4,11]. Viral vectors such as lentiviruses have also achieved high editing efficiencies in salmonid cell lines, but their integration into the host genome and sustained Cas9 expression raise biosafety concerns, and most fish cell lines are immune to lentivirus infection [4,6].

Lipofection methods, including those using lipid nanoparticles and lipofectamine reagents, have demonstrated moderate cellular uptake but limited editing efficiency in fish cells, often attributed to intracellular barriers such as endosomal retention and insufficient nuclear import; only nuclear localization correlates with gene editing outcomes [8,12,13]. For example, lipofection using Lipofectamine 3000 in CHSE/F cells yielded only ~10% transfection efficiency and no detectable genome editing [9,13]. Meanwhile, emerging delivery platforms, including magnetic nanoparticles and carbon nanotubes, are being explored in vivo in species such as *Poecilia reticulata* and *Litopenaeus vannamei*, but their application in marine fish cell lines is still limited [14]. As aquaculture continues to grow, there is a need to optimize CRISPR/Cas9 delivery methods tailored to specific cell types and species, consolidating fish cell lines as essential in vitro platforms for functional genomics and research on the genomic basis of traits of interest [10].

Magnetic nanoparticles, particularly superparamagnetic iron oxide nanoparticles (SPIONs), represent an emerging non-viral alternative that enables magnetically guided delivery while maintaining high biocompatibility. Their use in mammalian systems has shown enhanced transfection and editing efficiencies with low cytotoxicity, as demonstrated in porcine and human fibroblasts, and in patient-derived iPSC models [15,16,17,18]. Although not yet applied to marine teleost cell lines, these advantages justify their evaluation in species with low responsiveness to conventional transfection.

Another critical, yet often overlooked, factor influencing CRISPR/Cas9 performance is the aggregation behavior of the Cas9 protein. Protein aggregation can alter Cas9’s solubility, size, and interaction with delivery systems, potentially impairing encapsulation, uptake, and nuclear import. Recent studies have shown that buffer composition, pH, and gRNA binding can trigger Cas9 aggregation, increasing particle sizes from ~10 to over 200 nm [19,20,21]. This phenomenon may compromise delivery efficiency in both electroporation and nanoparticle-based systems, where size homogeneity and structural stability are essential for effective editing. Although direct evidence linking aggregation to editing outcomes is still limited, understanding this physicochemical barrier could improve the rational design of CRISPR delivery platforms.

In this study, we conducted a comprehensive comparative analysis of three CRISPR/Cas9 delivery strategies for genome editing in marine fish cell lines derived from European seabass (*Dicentrarchus labrax*) Brain 1 (DLB-1) and gilthead seabream (*Sparus aurata*) Brain 1 (SaB-1) (Grafical Abstract). The approaches included electroporation of Cas9–sgRNA ribonucleoproteins (RNPs), lipofection using sgRNA-loaded lipid nanoparticles (LNPs) with subsequent Cas9 internalization, and magnetofection with Cas9–sgRNA RNPs conjugated to fluorescent gelatin-coated superparamagnetic iron oxide nanoparticles (SPIONs@Gelatin). These nanoparticles were evaluated as a potential delivery alternative based on passive uptake and magnetic guidance.

We assessed not only transfection and editing efficiency, but also the subcellular localization of Cas9, tracked by Cy5 and FLUOGREEN labeling, and the genomic stability of the edited locus. In DLB-1 cells, multiple PCR amplicons suggested possible structural rearrangements that were absent in the original tissue source. Both in vitro transcribed and chemically modified sgRNAs (Synthego, Redwood City, CA, USA) were tested. This integrated evaluation provides a practical framework for optimizing genome editing protocols in non-model teleost species and highlights key factors such as nuclear import and genomic integrity that determine editing outcomes. Our findings support the application of these tools to advance aquaculture research and establish new platforms for the rational design of CRISPR delivery systems.

## 2. Results

### 2.1. Electroporation Efficiency

**Figure 1:**
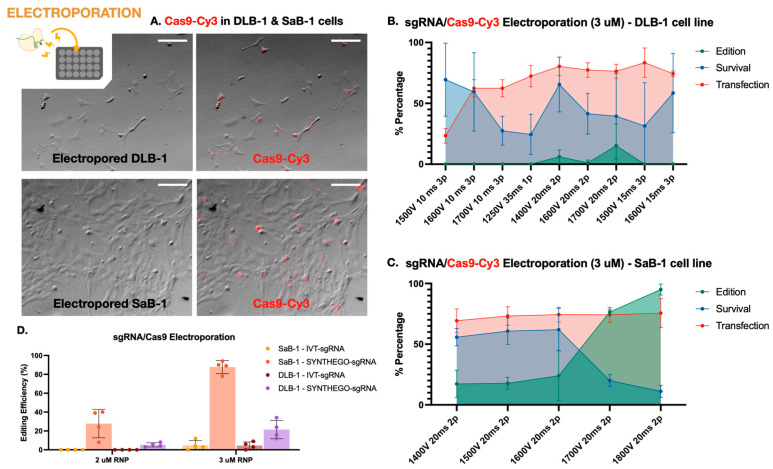
Electroporation-mediated genome editing in marine fish cell lines. (**A**) Fluorescence microscopy images of electroporated DLB-1 (**top**) and SaB-1 (**bottom**) cells transfected with Cy3-labeled Cas9. Images show brightfield and overlay with red fluorescence, indicating Cas9 uptake. SaB-1 cells displayed more widespread and intense fluorescence signal. Scale bar: 200 µm. (**B**) Transfection, survival, and editing percentages in DLB-1 cells after electroporation with 3 µM RNP under different voltage and pulse conditions. (**C**) Transfection, survival, and editing percentages in SaB-1 cells under different voltage and pulse conditions. (**D**) Editing efficiency in DLB-1 and SaB-1 cells using two different RNP concentrations (2 and 3 µM). SaB-1 cells consistently show higher editing rates, especially when using synthetic sgRNAs.

To characterize the performance of CRISPR-Cas9 editing in marine fish cell lines, we first systematically evaluated electroporation-based delivery of RNPs in DLB-1 and SaB-1 cells. This included assessing transfection efficiency, editing outcomes, and cell viability across a range of electroporation parameters and RNP concentrations. These results are summarized in Figure 1.

First, the best electroporation parameters in terms of transfection efficiency and cell viability were established in both cell lines. Transfection efficiency was evaluated using Cas9 protein conjugated to Cy3 (Cas9-Cy3), quantifying uptake 1 h post-electroporation. Cell viability was simultaneously assessed using trypan blue exclusion. Successful Cas9-Cy3 uptake was observed in both DLB-1 and SaB-1 cells, with SaB-1 displaying a more homogeneous and intense signal (Figure 1A). Quantifications performed under a fixed RNP concentration (3 µM) across different electroporation settings confirmed that SaB-1 cells maintained higher transfection efficiency and survival (Figure 1B,C). In contrast, DLB-1 cells showed more variability and sensitivity to electroporation parameters, with some conditions resulting in sharply reduced viability and transfection performance.

Based on this analysis, no single condition simultaneously maximized all variables in DLB-1 cells. The highest editing was obtained at 1700 V, 20 ms, and 2 pulses (up to ~28% editing), though this came at the expense of reduced viability. In contrast, 1600 V, 15 ms, 3 pulses provided a better compromise, yielding high transfection (~75%) and acceptable viability (~50%), albeit with moderate editing efficiency (~10%). For SaB-1 cells, editing efficiency increased with voltage, reaching nearly 100% at 1800 V, 20 ms, and 2 pulses, with high transfection (~78%) but lower survival (~20%). Intermediate conditions such as 1600 V offered a more balanced outcome, combining moderate editing with higher viability. These comparative results were used to select the optimal parameters for genome editing experiments.

Secondly, we aimed to evaluate the genome editing efficiency of a CRISPR-Cas9 RNP strategy targeting the *ifi27l2a* gene in both marine fish cell lines using the electroporation parameters tested above. We compared two *sgRNA* sources, in vitro transcribed (IVT) and chemically synthesized (Synthego), at two RNP concentrations (2 and 3 µM). For these experiments, electroporation was carried out under the conditions that previously yielded the highest editing efficiency at 3 µM RNP, as determined in Figure 1B,C: 1700 V, 20 ms, and 2 pulses for DLB-1 and 1800 V, 20 ms, and 2 pulses for SaB-1. As shown in Figure 1D, SaB-1 cells exhibited markedly higher editing frequencies than DLB-1, particularly when using synthetic sgRNAs at 3 µM, with editing reaching up to ~95%. In contrast, IVT sgRNAs resulted in lower or undetectable editing, especially in DLB-1. Testing a higher RNP dose (5 µM) in DLB-1 cells did not lead to further improvement and this condition was therefore not extended to SaB-1. Overall, these findings confirm that SaB-1 cells are more amenable to efficient genome editing than DLB-1, and that synthetic sgRNAs at 3 µM RNP under optimized electroporation conditions yield the most robust outcomes.

To better characterize the nature of the edits, indel profiles were analyzed using the ICE tool based on Sanger sequencing data. As shown in Supplementary Figure S1, DLB-1 cells exhibited a narrow mutation spectrum, with most edited alleles consisting of single-nucleotide insertions or small deletions around the sgRNA cut site. ICE analysis estimated that approximately 66% of the alleles remained unedited (Supplementary Figure S1A), indicating low editing efficiency and limited diversity. In contrast, SaB-1 samples showed a broader mutational landscape, including complex deletions and combined indels affecting both sgRNA target sites within the same locus (Supplementary Figure S1B). These two cut sites were simultaneously targeted using a pair of sgRNAs during electroporation. The most abundant edited allele accounted for 62% of the signal, while the remaining profiles consisted of numerous low-frequency indels, suggesting a more efficient and heterogeneous editing response in SaB-1 cells.

To evaluate whether the amount of Cas9–sgRNA RNP impacts editing efficiency, we quantified editing at two concentrations (2 and 3 µM) and calculated the corresponding RNP dose per cell. For SaB-1 cells, the most effective condition (3 µM Synthego RNP, ~94% editing) corresponded to ~0.0336 ng RNP per cell, while DLB-1 cells reached similar but modest editing levels at 3 µM (~30% editing), corresponding to ~0.0080 ng per cell (Figure 1D). Increasing the RNP dose to 5 µM in DLB-1 cells did not result in any substantial improvement in editing efficiency, and therefore, this condition was not further tested in SaB-1 cells. These per-cell estimates provide a functional context for the observed differences between cell lines, suggesting that intracellular RNP availability—rather than total protein uptake or sgRNA type alone—may act as a limiting factor for efficient genome editing in DLB-1 cells.

To examine whether intracellular Cas9 levels correlate with editing efficiency, we assessed Cas9-Cy5 uptake by flow cytometry across four protein concentrations (1–5 µM) using three representative electroporation protocols: 1400 V 20 ms 1 pulse, 1400 V 20 ms 2 pulses, and 1650 V 15 ms 2 pulses. These settings were selected based on previous viability and editing results (Figure 1B,C) to represent a range of moderate-to-high transfection conditions while capturing variability in pulse number and voltage. Mean fluorescence intensity (MFI) increased with protein dose in both DLB-1 and SaB-1, and SaB-1 cells consistently exhibited higher MFI values (Supplementary Figure S2A). However, the percentage of Cy5-positive cells remained close to 100% under all conditions, including non-electroporated controls, indicating that surface-bound or vesicle-trapped protein may contribute to total fluorescence (Supplementary Figure S2B). Among the tested electroporation settings, all showed similar uptake trends, but the 1400 V 20 ms 2-pulse condition produced the most reproducible and discriminative MFI response and was therefore selected for detailed statistical comparison (Supplementary Figure S2C). Under this condition, MFI increased significantly with concentration, but editing efficiency plateaued beyond 3 µM, suggesting that additional intracellular uptake does not translate into improved editing outcomes.

To visually inspect the cytotoxic effects of electroporation and Cas9-Cy5 loading, brightfield images of both cell lines were captured immediately prior to flow cytometry (Supplementary Figure S3A,B). Cells were subjected to increasing Cas9-Cy5 concentrations (0–5 µM) under four electroporation conditions. Morphological inspection revealed that higher voltages, pulse numbers, or protein concentrations were associated with visibly reduced cell density and signs of stress in both DLB-1 and SaB-1 cultures. These images confirmed the anticipated cytotoxic impact of more aggressive electroporation settings and provided qualitative validation of the viability trends quantified elsewhere.

Together, these results demonstrate that while electroporation can achieve high gene editing efficiency in SaB-1 cells, the DLB-1 line exhibits intrinsic limitations. In DLB-1, the highest editing efficiency (~30%) was obtained at 1700 V, 20 ms, 1 pulse, a condition that also resulted in moderate cell viability (~50%) (Figure 1B). In contrast, SaB-1 cells reached editing levels above 90% at 1800 V, 20 ms, 2 pulses, but this came at the cost of severely reduced survival (~10%) (Figure 1C). Further increases in voltage or RNP concentration in DLB-1 did not enhance editing efficiency, suggesting that intracellular trafficking and nuclear access may represent limiting steps in this cell line. Overall, while SaB-1 cells tolerate more aggressive electroporation and respond with efficient genome editing, DLB-1 cells appear fundamentally constrained by intrinsic aspects of their cellular physiology.

To investigate subcellular distribution, we performed confocal microscopy 24–48 h post-electroporation using Cas9 conjugated to Cy5 (Cas9-Cy5) (Supplementary Video S1). Confocal microscopy 24–48 h post-electroporation showed Cas9–Cy5 predominantly in the cytoplasm with perinuclear accumulations. Punctate, vesicle-like structures were evident in DLB-1. A diffuse, rounded perinuclear region was occasionally observed; however, without a nuclear counterstain we cannot confirm nuclear localization. We therefore describe the signal as mainly cytoplasmic with no definitive nuclear accumulation. In DLB-1, the signal appeared as punctate, vesicle-like structures, indicative of endosomal entrapment or restricted cytoplasmic diffusion. Complementary live-cell imaging of DLB-1 cells stained with Rhodamine123, which targets mitochondria, revealed persistent Cas9-Cy5 signal 48 h post-treatment (Video S1). The red fluorescence partially colocalized with cytoplasmic structures and also accumulated in a diffuse, rounded region lateral to the cell center, suggestive of potential nuclear localization. Additional discrete foci were visible throughout the cytoplasm, consistent with protein aggregation or vesicular sequestration.

Although no direct confocal analysis was conducted in SaB-1 under identical conditions, widefield imaging showed a more homogeneous cytoplasmic signal with fewer puncta. These observations suggest that inefficient cytosolic trafficking and limited nuclear access may contribute to the lower editing efficiency observed in DLB-1, compared to the more permissive behavior inferred in SaB-1.

### 2.2. Lipofection with Diversa LNPs: Promising Yet Suboptimal for Gene Editing

**Figure 2:**
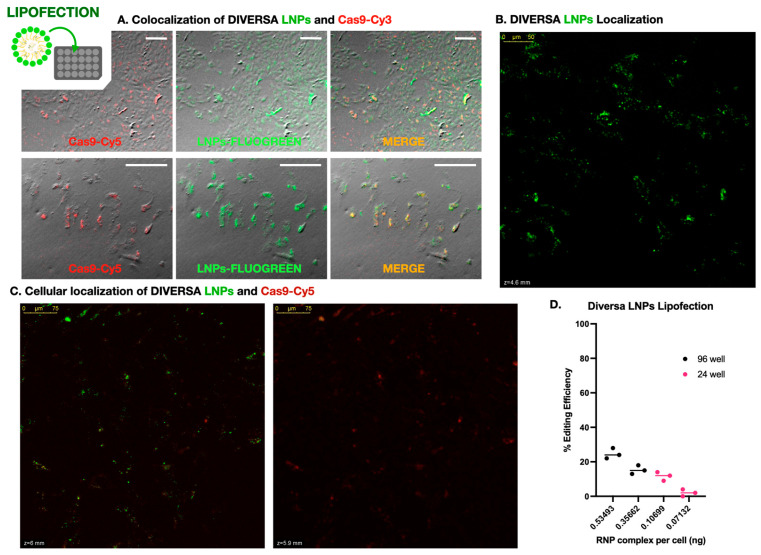
Lipid nanoparticle-mediated delivery of Cas9–sgRNA complexes in DLB-1 cells. (**A**) Widefield fluorescence microscopy showing colocalization of FLUOGREEN-labeled DIVERSA LNPs (green) and Cas9-Cy3 (red) in DLB-1 cells. Merged images confirm efficient co-delivery. Scale bar: 200 µm. (**B**) Confocal microscopy image of DLB-1 cells showing spatial distribution of FLUOGREEN-labeled DIVERSA LNPs 24–48 h post-transfection. The LNP signal is predominantly observed near the apical surface, indicating intracellular localization. (**C**) Confocal z-stack images showing differential localization of DIVERSA LNPs (green) and Cas9-Cy5 (red) in DLB-1 cells. LNPs accumulate near the apical region (z = 6 µm), while Cas9-Cy5 appears in deeper focal planes (z = 5.9 µm), suggesting cytosolic release after dissociation from LNPs. (**D**) Genome editing efficiency in DLB-1 cells using DIVERSA LNPs under different delivery conditions. Editing correlates with the estimated RNP complex dose per cell, calculated from fixed sgRNA concentration, reagent volume, and well format (96- vs. 24-well).

To address the poor genome editing efficiency observed in DLB-1 cells by electroporation, we explored lipid-based delivery systems as an alternative approach (Figure 2). As a first step, we evaluated whether DIVERSA lipid nanoparticles (LNPs) could successfully deliver Cas9–sgRNA complexes into cells. Using FLUOGREEN-labeled LNPs and Cy3-labeled Cas9, we performed widefield fluorescence microscopy and observed widespread green signals in nearly all cells, indicating robust association with the nanoparticles (Figure 2A). A substantial number of cells also showed clear red fluorescence (Cas9 presence), and merged images revealed colocalization between both signals, suggesting that Cas9 was co-delivered with the LNPs into the same cells. Although this initial assessment was qualitative, it strongly indicated that the lipid-based approach achieved a broader and more uniform delivery than electroporation. This interpretation was supported by confocal imaging of lipofected DLB-1 cells (Figure 2B), which showed intense FLUOGREEN signal concentrated near the cell periphery, suggesting widespread LNP-membrane interaction and efficient cell targeting.

To further investigate whether these signals corresponded to internalized material and to examine their relative subcellular localization, we performed confocal microscopy using Cas9 labeled with Cy5 (to avoid spectral overlap with the FLUOGREEN-labeled LNPs). Z-stack imaging acquired 24–48 h post-transfection revealed that the Cas9-Cy5 signal consistently appeared in focal planes deeper than the LNP signal, which remained closer to the apical surface of the cells (Figure 2B). This spatial separation is clearly visible in Supplementary Video S2, which highlights the axial displacement between both signals in a 3D projection. To quantify the z-separation in Supplementary Video S2, we re-analyzed the stack in Fiji (*Z*-axis profiles across 12 ROIs; peak slice per channel). We observed an apparent axial offset between LNP (green) and Cas9 (red) with a mean Δz = 16.42 ± 16.49 slices (median 17; range 0–41; N = 12 ROIs). Channels were acquired sequentially; no dye-swap or bead-based chromatic registration was performed, so minor chromatic aberration cannot be excluded. Accordingly, we report this as a descriptive observation. Supplementary Video S3, taken from a different region of the same culture, confirms this pattern and further includes an overlaid brightfield image, allowing clearer visualization of cell boundaries and reinforcing the intracellular localization of Cas9.

Although both videos illustrate the same distribution trend, their complementary focus—depth localization in Supplementary Video S2 and morphological context in Supplementary Video S3—justifies the inclusion of both. Moreover, Supplementary Video S4 provides a detailed view of an individual cell, showing the green LNP signal as more diffuse and confined to a superficial layer, while the red Cas9 signal is distributed at multiple points within the cytoplasm. These discrete red foci resemble the scattered Cas9 patterns observed in Supplementary Video S1, although no clear nuclear localization could be confirmed in this case, likely due to the use of culture plates with suboptimal optical quality, which limited resolution in the nuclear plane. Nonetheless, these results support successful transfection and intracellular delivery of Cas9.

We next investigated how genome editing efficiency was influenced by delivery conditions, focusing on reagent dose per cell. Four experimental setups were tested using identical LNP formulations (25 ng/µL sgRNA), applied at two volumes (50 µL and 75 µL) in two plate formats: 96-well (~20,000 cells/well) and 24-well (~100,000 cells/well). Since the total concentration of RNPs was fixed, the effective dose per cell varied depending on the cell density. Genome editing efficiency was highly dependent on the per-cell concentration of LNPs—i.e., exposure to Cas9–sgRNA complexes. As shown in Figure 2C, the highest editing efficiency (~25%) was obtained when 75 µL of LNPs was applied to a 96-well plate, corresponding to estimated amounts of 0.094 ng of sgRNA and 0.535 ng of RNP per cell. In contrast, the lowest efficiency (~2%) occurred in the 24-well plate with 50 µL of reagent, delivering only 0.013 ng of sgRNA and 0.071 ng of RNP per cell. These data clearly demonstrate that editing efficiency scales with the effective dose of RNPs per cell.

Importantly, the type of sgRNA used appeared to influence editing outcomes. In electroporation assays, chemically synthesized sgRNAs (Synthego) consistently outperformed in vitro transcribed (IVT) guides, confirming their superior performance in both cell lines. Although LNP-mediated delivery was primarily tested with synthetic sgRNAs, preliminary assays with IVT guides failed to produce detectable editing or yielded only very low efficiencies, possibly due to suboptimal dosing or reduced stability. Further optimization would be required to fully evaluate the compatibility of IVT sgRNAs with lipid-based delivery systems.

Assays were subsequently performed in SaB-1 under lipofection conditions matched to those optimized for DLB-1. However, editing efficiencies were consistently very low. Only one colony showed detectable indels (~5%) in 96-well plates at the highest sgRNA concentration, and no reproducible editing was observed across replicates. These results indicate that, under the tested conditions, SaB-1 is largely refractory to lipid-based transfection.

### 2.3. Cas9 Protein Aggregation

Previous experiments revealed the tendency of Cas9 to aggregate and its potential impact on the formation of the RNP complex, and consequently, on gene editing efficiency. For this reason, Cas9 aggregation was studied under various experimental conditions using Fluorescence Correlation Spectroscopy (FCS).

Throughout our comparative analysis of electroporation and lipid-based delivery strategies, we observed that Cas9 uptake alone does not necessarily translate into effective genome editing. Flow cytometry revealed high percentages of Cy5-positive cells even under non-electroporated conditions—suggesting potential membrane association or passive surface adsorption of Cas9. Moreover, confocal microscopy consistently showed heterogeneous and predominantly cytoplasmic distribution of the protein across delivery conditions, with limited nuclear localization regardless of the method used. This was especially evident in lipid-mediated delivery, where Cas9 and nanoparticles occupied distinct intracellular compartments.

These observations raised the possibility that the physicochemical state of Cas9 itself—particularly its tendency to aggregate—might be influencing its delivery efficiency, intracellular distribution, and ultimately its genome editing activity. This hypothesis was further supported by the apparent discrepancy between intracellular Cas9 levels and functional editing outcomes observed across several experimental conditions. We therefore sought to evaluate whether differences in aggregation behavior could explain, at least in part, the discrepancies observed between delivery methods.

FCS curves (Supplementary Figure S4) were obtained for Cas9 labeled with a cyanine dye (see the Methods and Materials section) under the experimental conditions of two of the delivery strategies, electroporation and lipofection with Diversa LNPs, to try to assess the conditions of the Cas9 molecule and relate it with their editing efficiency. The conditions chosen for the different delivery methods were the following:

- For electroporation, the labeled Cas9 was measured in cell culture medium, which is the environment in which Cas9 is present during the electroporation process.
- For the lipofection with Diversa LNPs, the final step of the preparation protocol was selected as a key point in the delivery process. In this step, the preloaded-sgRNA LNPs are incubated with Cas9 dissolved in a 10 mM HEPES solution at pH 7.4. Therefore, the Cas9 was measured in this buffer solution.

The curves were analyzed to obtain a diffusion correlation time, $t_{D}$, related to the size and shape of the particle. Diffusional properties like the diffusion coefficient, D, and hydrodynamic radius, $R_{H}$, were calculated from $t_{D}$ (see the Methods and Materials section) and shown in Table 1.

We observe a much larger diffusion time for Cas9 in the lipofection buffer in comparison to the Cas9 in the cell medium used in electroporation assays. This difference suggests that Cas9 tends to form higher-order aggregates or multimeric species in lipofection buffer, which dissolve in the presence of salt and other compounds present in the cell medium. This may allude to a potential aggregation tendency of Cas9 alone in solution, which could alter its intracellular behavior after delivery. Some authors have already reported Cas9 aggregation under similar buffer conditions [19,20,22].

The diffusion coefficient and hydrodynamic radius of the Cas9 monomer, observed in cell medium, align well with previously reported values in the literature, which place the size of monomeric Cas9 in 10.3 ± 1.5 nm, obtained with Dynamic Light Scattering techniques [19,20].

The hydrodynamic radius of Cas9 in HEPES is nearly twice that of the monomeric form due to aggregate formation. This could interfere with its internalization into the Diversa LNPs in the incubation step of Diversa’s protocol, just before the gene editing process.

These findings underscore the importance of molecular characterization of Cas9 formulations under different delivery conditions, as aggregation could significantly influence editing outcomes—even in cases where intracellular uptake is confirmed. An aggregation study on the Cas9 protein is currently underway by our group.

### 2.4. Evaluation of CRISPR-Cas9 Magnetofection Using Iron Oxide Nanoparticles Coated with Gelatin

To evaluate intracellular barriers limiting CRISPR/Cas9 delivery in marine fish cell lines, we tested a magnetofection approach based on gelatin-coated superparamagnetic iron oxide nanoparticles (SPIONs@Gelatin) conjugated with Cas9–sgRNA RNPs. RNPs were assembled in vitro by complexing recombinant Cas9 protein with synthetic sgRNAs (Synthego), followed by bioconjugation onto FITC-labeled SPIONs using EDC/Sulfo-NHS chemistry. These complexes were applied to DLB-1 and SaB-1 cells under magnetofection conditions, with or without exposure to an external magnetic field generated by custom neodymium magnet plates. Doses of 100, 200, and 500 µL per well (equivalent to 0.41, 0.82, and 2.05 ng of RNP per cell) were tested at two incubation times (30 and 60 min).

**Figure 3:**
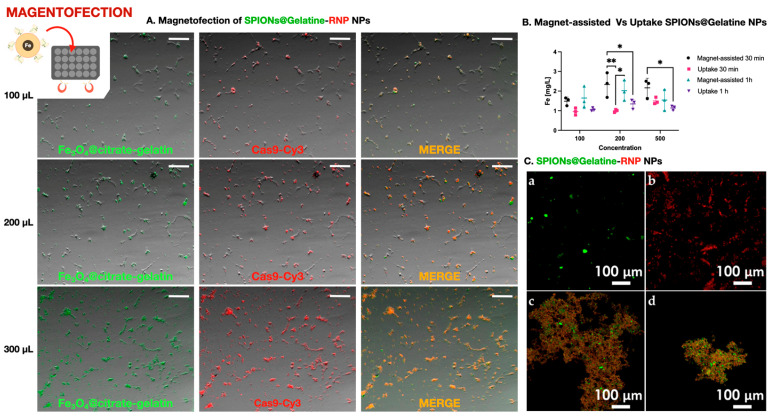
Magnetofection-mediated delivery of Cas9 using SPIONs@Gelatine nanoparticles. (**A**). Fluorescence microscopy images showing co-delivery of SPIONs@Gelatine nanoparticles (green) and Cas9-Cy3 (red) in cells treated with 100, 200, or 300 µL of nanoparticle suspension. Merged yellow/orange signal indicates intracellular colocalization. Scale bar: 200 µm. (**B**). Quantification of iron content in cells following passive incubation (Uptake) or magnet-assisted delivery (Magnet-assisted) for 30 min or 1 h, using SPIONs at 100, 200, or 500 µL. A statistically significant increase in iron internalization was observed under magnetic conditions, especially at 200 µL after 30 min, as determined by two-way ANOVA followed by Tukey’s multiple comparisons test (* *p* < 0.05, ** *p* < 0.01; n = 3). (**C**). Confocal microscopy images showing (**a**) FITC-labeled SPIONs@Gelatin, (**b**) Cy3-labeled Cas9–sgRNA RNPs alone, and their respective bioconjugated complexes prepared for genome editing of the *ifi27l2a* gene in marine fish cell lines. Panels (**c**) and (**d**) correspond to the RNPs specific for DLB-1 and SaB-1 cells, respectively, conjugated to SPIONs@Gelatin prior to delivery. The sgRNAs used were designed based on the *ifi27l2a* sequence of each species.

Fluorescence microscopy showed robust delivery of SPIONs@Gelatin–RNPs, with dose-dependent increases in green (FITC-labeled nanoparticles) and red (Cas9-Cy3) signals (Figure 3A). Colocalization of both signals (yellow/orange) indicated stable conjugation and cell-associated retention. These signals persisted after multiple washes, supporting stable association with the cell surface or interior. However, due to the resolution limitations of widefield microscopy, we cannot exclude the possibility that some fluorescent signals derive from membrane-associated aggregates rather than fully internalized complexes.

To quantify nanoparticle internalization without interference from protein–nanoparticle interactions or fluorescence overlap, we used SPIONs@Gelatin nanoparticles without RNPs. As shown in Figure 3B, intracellular iron levels increased with nanoparticle concentration and were significantly higher in magnet-assisted conditions, particularly at 200 µL after 30 min. Statistical analysis by two-way ANOVA followed by Tukey’s post hoc test confirmed significant differences between uptake conditions (* *p* < 0.05, ** *p* < 0.01; n = 3). These results validate that magnetofection effectively enhances cellular internalization of SPIONs under the same physical and temporal conditions used for RNP delivery.

Prior to their application, bioconjugated SPIONs@Gelatin–RNP complexes were validated by confocal microscopy to confirm successful labeling and conjugation. As shown in Figure 3C, FITC-labeled nanoparticles, Cy3-labeled RNPs, and the resulting conjugates specific for DLB-1 and SaB-1 cells exhibited distinct and overlapping fluorescent signals, confirming efficient complex formation for genome editing of the *ifi27l2a* gene.

Despite efficient uptake and colocalization of Cas9 and nanoparticles, PCR-based genotyping consistently failed to detect genome editing in either cell line across all tested conditions. Two independent experiments, each with six biological replicates per condition, revealed no detectable indels at the target locus. Occasional low-frequency editing signals (<4%) were sporadically observed in isolated replicates, but lacked reproducibility or correlation with experimental variables, and were thus considered background artifacts.

Notably, our SPIONs were not specifically optimized for nuclear targeting, which may partially explain the absence of editing despite effective delivery. Even at the lowest nanoparticle dose (0.41 ng of RNP per cell), the amount was comparable to the most efficient Diversa LNP condition in DLB-1 cells (0.535 ng/cell), while the highest dose (2.05 ng/cell) exceeded it nearly fourfold. Yet, none of the magnetofection conditions led to detectable editing, underscoring that intracellular accumulation alone is insufficient. Similarly to results obtained with Diversa LNPs, where delivery of Cas9 did not always result in editing unless specific criteria were met, these findings highlight that critical intracellular barriers—such as endosomal escape, RNP release, and nuclear import—likely limit editing efficiency.

Altogether, our results indicate that although SPIONs@Gelatin–RNP complexes are robustly internalized and retained by marine fish cells under magnetofection conditions, their inability to mediate genome editing suggests that post-uptake bottlenecks must be addressed. Optimizing strategies for endosomal escape and nuclear trafficking is essential to unlock the full potential of nanoparticle-mediated CRISPR delivery in hard-to-transfect systems.

### 2.5. Amplification and Sequencing

Amplification and sequencing of the *ifi27l2a* gene revealed striking differences in the genomic architecture of DLB-1 and SaB-1 fish cell lines. Early PCR attempts on DLB-1 genomic DNA using standard Taq polymerases yielded weak, diffuse, or inconsistent bands, in contrast to the cleaner and more robust amplification observed in SaB-1 cells and seabass tissue gDNA. As shown in Supplementary Figure S5A, amplification quality was influenced by both the DNA extraction method and the polymerase used. In general, Chelex 100 (Bio-Rad, Hercules, CA, USA) and DNeasy (Qiagen, Hilden, Germany) protocols outperformed QuickExtract (Lucigen, Middleton, WI, USA), especially when combined with BioTaq (Bioline, Cincinnati, OH, USA) or AmpliTaq Gold (Applied Biosystems, Waltham, MA, USA). BioTaq consistently produced the most intense amplification products but also generated additional non-specific bands across all three primer pairs (PE, P1, P2), particularly in DLB-1 (highlighted with red asterisks). AmpliTaq Gold offered a balance between intensity and specificity, producing cleaner results with fewer spurious bands; GoTaq (Promega, Madison, WI, USA) showed intermediate performance. Across all polymerases and extraction methods, the seabass tissue control yielded a single specific band for PE, P1, and P2, whereas multi-band products appeared only for the P2 target in cell-line templates (SaB-1, DLB-1). Sanger sequencing of the single-band products confirmed the expected *ifi27l2a* sequence (see Supplementary Figure S5, sequencing panel).

Guide design and in silico specificity metrics are summarized in Supplementary Table S1. For each sgRNA we report two classes of information: (i) specificity, captured by MIT and CFD scores together with the total number of predicted off-targets; and (ii) on-target behavior, summarized by Doench’16 and Azimuth activity scores plus frameshift-oriented predictors (Out-of-Frame and Lindel) and the Moreno-Mateos score. To aid interpretation, off-targets are stratified by mismatch class (0–1–2–3–4) and, on the line below, by the PAM-proximal ‘next-to-PAM’ subset (seed region). In seabass, DLB1-sgRNA1 shows 0–2–11–34–277 total predictions with 0–2–2–6–2 in the seed region, and DLB1-sgRNA2 shows 0–3–4–13–172 totals with 0–0–0–0–1 seed-proximal. Thus, only two 1-mismatch seed-proximal sites are predicted for sgRNA1 and none for sgRNA2, whereas the remaining predictions are skewed to ≥2 mismatches. The seabream guides present even fewer candidates. This distribution indicates that high-risk, PAM-proximal off-targets are rare in our designs.

Despite these optimizations, PCR amplification of the *ifi27l2a* locus from DLB-1 cells frequently yielded multiple bands, especially with the P2 primer pair, regardless of the polymerase or extraction method used. These additional bands, which were absent in SaB-1 and seabass DNA, are indicated by red asterisks in Supplementary Figure S5A and likely reflect underlying genomic alterations in the DLB-1 cell line. In contrast, both SaB-1 cells and crude seabass DNA consistently produced a single, sharp product with each primer set, consistent with an intact and non-rearranged target locus.

Sequencing of the P2 amplicon from crude seabass DNA confirmed the presence of a single canonical exon. Sanger chromatograms showed high-quality, unambiguous reads with no evidence of mixed sequences or allelic variation (Supplementary Figure S5B). These results suggest that the multiple PCR products observed specifically in DLB-1 cells are not due to primer misannealing or polymerase error but rather reflect culture-acquired genomic duplications or structural rearrangements at the target locus. These findings are consistent with previous reports of chromosomal instability in long-term fish cell cultures. Importantly, the presence of duplicated or rearranged genomic regions could interfere with CRISPR-Cas9 genome editing by introducing multiple near-identical target sites, thereby reducing on-target precision or increasing off-target activity. Conversely, the consistent single-band amplification and clean exon sequence observed in SaB-1 support its use as a genomically stable and tractable platform for functional genomics and precise genome editing in marine fish models.

To confirm these observations and improve amplification consistency, we tested a high-fidelity proofreading enzyme, Platinum™ SuperFi™ II DNA polymerase (Thermo Fisher Scientific, Waltham, MA, USA), on the same DNA samples and target regions (PE, P1, P2). As shown in Supplementary Figure S6, this polymerase produced sharp, specific bands across all extraction methods, including QuickExtract, and minimized non-specific amplification. Accordingly, enzyme-dependent negatives (e.g., Kit–PE with Applied) most likely reflect polymerase sensitivity to residual inhibitors rather than loss of the target, consistent with the uniform amplification obtained with SuperFi II. However, even under these optimized conditions, the P2 amplicon from DLB-1 still displayed banding patterns distinct from those of SaB-1 or seabass tissue, suggesting that the observed heterogeneity arises from genuine genomic alterations rather than enzyme artifacts. These results reinforce the conclusion that DLB-1 cells harbor culture-acquired rearrangements at the *ifi27l2a* locus, which may compromise PCR-based analyses and gene-editing assays.

## 3. Discussion

This study provides a rigorous comparative assessment of three CRISPR/Cas9 delivery systems: electroporation, lipid-based transfection using Diversa LNPs, and magnetofection using SPIONs@Gelatin–RNPs, in two marine fish cell lines (DLB-1 and SaB-1) targeting *ifi27l2a*. This gene has been identified as a strong candidate for resistance to nervous necrosis virus (NNV), particularly in European sea bass, based on QTL and eQTL analyses [23,24,25]. Our comparison was scoped around pre-assembled Cas9–sgRNA RNPs (electroporation, magnet-assisted uptake) and LNPs formulated to deliver sgRNA with subsequent Cas9 internalization. We prioritized RNPs because they provide immediate activity without a translation step and shorten the exposure window of active Cas9. Cas9 mRNA plus sgRNA is a viable alternative in LNP/nucleofection contexts and may extend editing duration when translation is efficient; testing this modality in these teleost lines is a logical next step but was outside the scope of the present study.

### 3.1. Electroporation Enables Efficient Genome Editing in Marine Fish Cell Lines

Electroporation yielded the highest editing performance under our conditions. As a physical delivery method, it bypasses endocytic trafficking and permits direct cytoplasmic entry, thereby facilitating nuclear import of Cas9 [12,21]. This is consistent with Qin et al. (2016), who achieved efficient editing in fish embryos using plasmid-encoded ZFNs delivered by electroporation [26].

In contrast, editing in DLB-1 cells was markedly less efficient despite similar uptake of Cas9-Cy5, as confirmed by flow cytometry and microscopy. Notably, PCR amplification of the *ifi27l2a* locus in DLB-1 yielded multiple amplicons, whereas genomic DNA from the original fish source produced a single band. This strongly suggests structural variation in the cell line, such as gene duplication, partial rearrangement, or chromosomal fragmentation, phenomena frequently observed in long-term cultured teleost cells [13,27,28]. Such instability can hinder CRISPR by introducing competing target sites, altering PAM accessibility or disrupting allelic homology; high-quality reads indicate that these anomalies reflect genuine genomic complexity rather than artifacts. Consistent with this interpretation, the P2-restricted multi-band readout seen only in cultured cells (Supplementary Figure S5A) dovetails with structural heterogeneity at *ifi27l2a* in DLB-1, which can dilute or misdirect RNP activity and help explain lower or more variable editing compared with SaB-1.

A potential additional contributor to the lower editing in DLB-1 is guide sequestration by off-target binding/cleavage. However, our in silico profiling (Supplementary Table S1) indicates that the predicted off-targets are heavily skewed toward ≥2 mismatches and/or non-canonical PAMs: for the two seabass guides used here, only 2 (sgRNA1) and 3 (sgRNA2) NGG 1-mismatch candidates are predicted, with very few high-risk PAM-proximal (seed) sites (0–1 per guide). Thus, while off-target activity cannot be excluded and will be assessed by targeted sequencing of the top-ranked sites, widespread off-target cleavage is unlikely to be the dominant cause of the low DLB-1 editing compared with the locus-level heterogeneity documented by PCR and sequencing.

In both lines, chemically synthesized sgRNAs outperformed IVT sgRNAs at matched molar doses. The synthetic guides carried 2′-O-methyl and 3′-phosphorothioate modifications at the first and last three nucleotides, which likely improve stability against nucleases and maintain RNP integrity during and after electroporation. These chemical features, rather than differences in loading or dosing, best explain the higher editing observed with synthetic sgRNAs in our setting. In DLB-1, we co-targeted two adjacent cut sites with a 1:1 sgRNA mix; single-guide or wider-spacing designs were not tested here and are practical routes to broaden the mutational spectrum in future optimization.

### 3.2. LNP-Mediated Delivery Achieves Transfection but LIMITED Editing in DLB-1 Cells

Diversa LNPs enabled efficient delivery of Cas9 RNPs into DLB-1 cells, but genome editing remained modest. In SaB-1, under identical conditions, editing efficiency was even poorer, with only a single instance of ~5% editing detected, highlighting the limited applicability of lipofection in this line. This discrepancy likely arises from a combination of intrinsic cell line characteristics and potential formulation-related factors.

FCS indicated that Cas9 tends to aggregate in the HEPES buffer used during the incubation step of the Diversa protocol, potentially reducing its ability to form stable RNP complexes and limiting effective encapsulation within LNPs. This aggregation could also hinder intracellular release and nuclear import, as suggested by the punctate cytoplasmic fluorescence observed in DLB-1 cells (Supplementary Videos S2–S4). This pattern is consistent with previous observations of electroporated Cas9, which also exhibited punctate staining, potentially indicative of aggregation, endosomal entrapment, or other forms of intracellular sequestering [12].

In addition to these observations, recent studies have highlighted the aggregation state of Cas9 as a critical physicochemical factor that may impair genome editing efficiency. Protein aggregation can reduce Cas9 solubility, alter its size, and disrupt interactions with sgRNA or delivery vehicles. For instance, Cas9–gRNA complexes can expand to ~200 nm depending on buffer composition and pH, while electroporated Cas9 has been shown to accumulate in cytoplasmic foci lacking nuclear access [12,19,20]. Although the direct causal link between aggregation and editing failure remains underexplored, these findings suggest that Cas9 aggregation may be a key barrier limiting LNP-mediated delivery in fish cells.

Importantly, the RNP doses used in these assays reflect the total amount of complex added to the cultures, not the actual quantity internalized or reaching the nucleus, which remains challenging to quantify. This distinction is critical, as the efficiency of intracellular delivery and the fraction of RNP that successfully reaches the nucleus remain largely unquantified [4,11,12,13,29,30]. Additionally, these calculations are based on the initial loading amounts, without direct measurement of intracellular RNP levels, meaning that the reported efficiencies are normalized to the total complex used, not necessarily the amount that actually penetrated the cells or reached the nucleus. This is a significant limitation, as variations in uptake efficiency, endosomal escape, and nuclear import can dramatically influence the final editing outcomes Goodwin and Huang, [31].

### 3.3. Iron Oxide Nanoparticles Enable Uptake but Not Functional Editing

Magnetofection using SPIONs@Gelatin resulted in strong Cas9-Cy5 signal in both DLB-1 and SaB-1 cells, yet no genome editing was detected under any tested condition. Although Cas9 fluorescence was clearly visible in the cytoplasm, no nuclear localization was observed, and PCR-based genotyping consistently failed to detect indel formation. These findings suggest that, despite uptake, the RNPs were likely trapped within endosomal compartments or degraded prior to reaching the nucleus. Specifically, multivalent adsorption of Cas9–sgRNA onto the SPION–gelatin surface may sterically shield NLSs and delay RNP release, while the lack of pH-responsive or fusogenic elements limits endosomal escape and timely cytosolic availability. This limitation is not unique to our system—similar challenges have been reported in mammalian cells using nanoparticle-based CRISPR delivery platforms that lack mechanisms for endosomal escape or nuclear targeting [32]. Unlike virus-like particles (VLPs), which can be engineered to bypass these barriers, the iron oxide particles used here do not possess fusogenic elements or targeting ligands. Although ineffective for DNA editing, these nanoparticles may still serve as valuable tools for decoupling uptake from functional delivery or for transporting molecules with lower nuclear access requirements, such as RNA-targeting enzymes.

Fluorescence microscopy further supported these conclusions by showing persistent retention of the RNP–nanoparticle complex in the cell layer up to 48 h post-treatment. Both Alexa-labeled gelatin (green) and Cas9-Cy3 (red) signals increased in intensity with nanoparticle dose and magnetic enhancement, and their merged colocalization confirmed that Cas9 remained stably conjugated to the particles over time. This pattern is consistent with endosomal sequestration and/or lysosomal routing rather than productive nuclear delivery, reinforcing that uptake alone is insufficient for editing.

Fluorescence correlation spectroscopy (FCS), used in this study to characterize aggregation of Cas9 in electroporation and LNP buffers, could not be applied under magnetofection conditions. As described in Materials and Methods, the SPIONs@Gelatin complexes were prepared in water, and their large size and strong fluorescence intensity fall outside the detection limits of the technique. Moreover, the aggregation of multiple Cas9–RNP units on the nanoparticle surface generates a highly polydisperse system that cannot be analyzed as freely diffusing individual molecules. As a result, FCS-derived diffusion parameters cannot be meaningfully compared across delivery systems, which limits direct physicochemical insight for this condition.

### 3.4. Editing Depends on Nuclear RNP Dose and Cell-Specific Trafficking

Across platforms, editing tracked with the nuclear availability of functional, aggregate-free Cas9 RNPs rather than total cellular uptake. Electroporation consistently resulted in the highest editing efficiencies, particularly in SaB-1 cells, where >90% indel formation was achieved with an estimated delivery of ~0.017 ng Cas9 and ~0.0080 ng sgRNA per cell. In contrast, both lipid-based (Diversa LNPs and Lipofectamine 3000) and iron nanoparticle-based methods delivered higher amounts of RNPs per cell in DLB-1—~0.535 ng Cas9 in the case of Diversa LNPs—but failed to produce detectable editing. This supports the conclusion that total uptake is insufficient: successful editing requires that a threshold concentration of RNPs be reached in the nucleus, a finding consistent with [12], who demonstrated via FCS that efficient genome editing in mammalian cells requires ≥1300 RNPs per nucleus and prolonged nuclear residency.

As described for LNPs above, aggregation-prone conditions are compatible with vesicular retention and poor nuclear trafficking. Notably, no editing was observed in conditions with strong uptake but absent nuclear localization, such as Lipofectamine 3000 in DLB-1 (Supplementary Figure S7), or magnetofection of iron nanoparticles in either line. This reinforces the notion that nuclear access is the critical bottleneck. At the same time, nuclear delivery alone is not sufficient: the P2-specific multi-band pattern in DLB-1 (Supplementary Figure S5A) indicates locus-level heterogeneity at *ifi27l2a* that can undermine editing even when cellular uptake is high. This dissociation between fluorescence signal and editing outcome underscores the need to complement transfection metrics with functional assays and to avoid over-reliance on fluorescence intensity as a proxy for editing efficiency. Consistently, the specificity and efficiency scores in Supplementary Table S1, together with the mismatch and seed-region breakdown, argue against a dominant contribution of widespread off-target activity in DLB-1.

Future studies could incorporate dual-color imaging of sgRNA and Cas9 to refine the analysis of RNP subcellular dynamics. However, as sgRNA lacks nuclear localization signals and depends on Cas9 for nuclear entry, fluorescent tracking of Cas9 alone provides functionally relevant information on editing efficiency [33].

### 3.5. Future Directions

The development of alternative delivery platforms continues to expand the toolbox for gene editing in aquatic species. In practical terms, our results prioritize two innovation paths for marine teleost cells: (i) adapting viral vectors, such as lentiviruses and baculoviruses when stable CRISPR expression is required, and (ii) engineering non-integrative carriers for RNPs. Viral vectors such as lentiviruses and baculoviruses have been successfully used to transduce fish cells and may allow for stable expression of CRISPR components [3,34]. Likewise, virus-like particles and pseudotyped systems represent a non-viral route for delivering Cas9 RNPs that natively bypass vesicular barriers and are therefore promising for marine species [32]. Both strategies directly target the dominant bottlenecks highlighted in our study: aggregation, endosomal sequestration and nuclear exclusion, thus offering a clear, testable route to more efficient delivery in marine bony fish [12,13].

Ultimately, effective genome editing in marine fish cells requires coordinated optimization of the delivery system, RNP formulation, nuclear import mechanisms, and the structural integrity of the target genome. Our results support electroporation as the most robust and reproducible technique for functional editing. However, the identification of structural alterations at the *ifi27l2a* locus in DLB-1 cells, reflected in the presence of multiple PCR amplicons and inconsistent editing outcomes, raises concerns about the reliability of editing readouts in cell lines with complex genomic rearrangements. These observations are consistent with the findings of Gonçalves et al. (2019), who showed that CRISPR-Cas9 targeting of tandemly duplicated or rearranged regions can trigger gene-independent loss-of-fitness effects [35]. Accordingly, routine structural checks (karyotyping, long-read sequencing or copy-number metrics) should accompany functional assays to validate targets and interpret outcomes.

The establishment of gene-edited marine fish cell lines, such as those targeting *ifi27l2a*, offers a valuable platform to investigate antiviral responses and host–pathogen interactions under controlled in vitro conditions. These systems reduce the need for in vivo experimentation and facilitate functional validation of immune-related genes, as demonstrated by the robust editing achieved in SaB-1 through electroporation. Consequently, optimization efforts were concentrated on DLB-1 cells, whose consistently lower performance and technical challenges provided a more informative model to explore alternative delivery strategies.

Gene-edited cell lines also support broader applications, including toxicological assays, infection challenges, and pre-validation of CRISPR reagents, which are increasingly relevant for sustainable aquaculture research [21,31,32,36]. As delivery systems and functional assays continue to evolve, genome editing in fish cell lines stands as a promising tool for mechanistic studies, biomarker discovery, and translational applications in aquatic biomedicine. Gene-specific functional assays were not included in this delivery-focused comparison and will be addressed in follow-up studies.

## 4. Materials and Methods

### 4.1. Cell Culture

DLB-1 (*D. labrax*) and SaB-1 (*S. aurata*) cell lines were maintained under optimized conditions to ensure stable proliferation and viability. Cells were cultured in Leibovitz’s L-15 medium (Thermo Fisher Scientific), supplemented with 10% fetal bovine serum (FBS) and 1% penicillin/streptomycin (P/S). Cultures were maintained at 28 °C in a CO_2_-independent environment. Cells were routinely passaged at 80–90% confluence using 0.25% trypsin-EDTA solution, incubated for 2–3 min at room temperature. Trypsin activity was neutralized with complete medium containing FBS, and cells were then resuspended and replated at a controlled density. All experiments were conducted using cells at no more than 15 passages to minimize genomic instability.

DLB-1 and SaB-1 cell lines were originally derived from brain tissue of European sea bass and gilthead seabream, respectively, and were established at the University of Murcia using retroviral immortalization with the snakehead retrovirus (SnRV), as described in Morcillo et al. (2017) and Ruiz-Palacios et al. (2020) [37,38]. Both cell lines have been genetically and functionally characterized, showing glial marker expression and susceptibility to viral infection. They are registered in the Cellosaurus database under the following accession numbers: DLB-1 (CVCL_HG31) and SaB-1 (CVCL_A9H7).

### 4.2. Primer Design for PCR Amplification of ifi27l2a

To amplify the *ifi27l2a* locus in DLB-1 and SaB-1 cells, multiple primer pairs were designed using NCBI Primer-BLAST (https://www.ncbi.nlm.nih.gov/tools/primer-blast/, accessed on 12 April 2024) based on the reference sequence of *D. labrax*. Three independent amplicons of 855 bp, 630 bp, and 419 bp were selected to ensure robust amplification and facilitate sequencing across the target region. In parallel, species-specific primer pairs were also designed for SaB-1 using the *S. aurata ifi27l2a* ortholog, yielding amplicons of 480 bp and 593 bp suitable for genotyping edited clones. Primer selection prioritized specificity, optimal melting temperature (Tm), GC content, and low self-complementarity. The sequences and thermodynamic parameters of all primers are detailed in Supplementary Table S2.

### 4.3. sgRNA Design, Synthesis and Preparation

Two sgRNAs targeting *ifi27l2a* were selected per species or use in the corresponding cell lines (DLB-1 and SaB-1), based on predicted on-target activity and minimal off-target risk using CRISPRscan and genome annotations. The guide sequences, together with the T7 promoter–containing forward primers and the universal reverse oligo used for template generation, are listed in Supplementary Table S3. Guides were designed with CRISPRscan (to prioritize on-target activity) and evaluated with CRISPOR against the corresponding species assemblies (SpCas9, NGG PAM). We report MIT and CFD specificity scores, the total number of predicted off-targets, and on-target/frameshift predictors (Doench’16, Azimuth in vitro, Moreno-Mateos, Out-of-Frame, Lindel). Off-targets are summarized by mismatch class (0–1–2–3–4) and by the PAM-proximal “next-to-PAM” seed-region subset; high-priority candidates for subsequent validation are defined a priori as NGG 1-mismatch, seed-proximal sites (if present).

Templates for in vitro transcription (IVT) were generated by PCR amplification using the T7 forward primer and the universal reverse oligo. PCR products (~117 bp) were purified using a silica-based column kit. IVT was performed using the MAXIscript™ T7 Transcription Kit (Thermo Fisher Scientific), and the resulting RNA was treated with DNase I, precipitated with LiCl and ethanol, washed, and resuspended in RNase-free water. RNA integrity was verified by gel electrophoresis, and concentration was measured with a NanoDrop spectrophotometer.

Additionally, chemically synthesized sgRNAs corresponding to the selected sequences were obtained from Synthego (Redwood City, CA, USA). These were diluted to working concentrations (50–100 nM) in nuclease-free water and stored at –80 °C until use. Both IVT and synthetic sgRNAs were tested in parallel to compare genome editing efficiency.

### 4.4. Electroporation

To deliver the CRISPR-Cas9 complex into the cells, electroporation was performed using a protocol optimized for marine fish cell lines. Cells were harvested at 80–90% confluency, washed with phosphate-buffered saline (PBS), and centrifuged at 600× *g* for 5 min. The pellet was resuspended in Opti-MEM (Gibco, Waltham, MA, USA) to a final density of 1 × 10^6^ cells per reaction. In parallel, Cas9 protein (IDT) was precomplexed with chemically synthesized sgRNA (Synthego, containing 2′-O-methyl and 3′-phosphorothioate modifications at the first and last three nucleotides) at a 1:2 molar ratio to form RNP complexes. The same molar concentrations were used when assembling RNPs with in vitro transcribed sgRNAs, ensuring equivalence between both sgRNA types for all comparative assays. After 10–15 min of incubation at room temperature, the RNP mix was combined with the cell suspension in a total volume of 10 µL for electroporation.

Cells were electroporated using different conditions depending on the assay. For the optimization experiment, electroporation was performed using 3 µM RNP under nine or five distinct combinations of voltage, pulse number, and pulse duration depending on the specie (as shown in Figure 1B,C). For the dose–response assay, SaB-1 cells were electroporated with 1 and 3 µM RNP, and DLB-1 cells with 1, 3, and 5 µM RNP using the condition determined as optimal in the previous experiment. Immediately after electroporation, 10 µL of the mixture were transferred into wells of a 24-well plate containing 990 µL of complete L-15 culture medium (supplemented with 10% fetal bovine serum and 1% glutamine, but without antibiotics). Cells were incubated at 25 °C, and after 24 h, the medium was replaced with fresh L-15 containing antibiotics. Cells were incubated at 25 °C, and after 24 h, the medium was replaced with fresh medium containing antibiotics.

To evaluate transfection efficiency shortly after delivery, replicate electroporations were carried out using Cas9 protein labeled with Cy3 (Cas9-Cy3). Immediately after electroporation, 10 µL of the cell–RNP suspension were added to each well of a 24-well plate containing 990 µL of complete L-15 medium (10% FBS, 1% GlutaMAX, no antibiotics; final volume 1 mL per well). After 1 h of incubation at 25 °C, when cells were still in suspension and had not adhered to the culture surface, 10 µL of cell suspension was collected from each well and mixed 1:1 with 0.4% Trypan Blue Stain (Cat# 15250-061, Gibco, Thermo Fisher Scientific). The mixture was gently homogenized and loaded into a Neubauer chamber. Manual counts of fluorescent (Cy3-positive) and non-fluorescent cells were performed under widefield fluorescence microscopy. Cell viability was assessed simultaneously by trypan blue exclusion. This sampling step did not affect total cell recovery, and no reduction in cell number or viability was observed.

For electroporation, RNPs were assembled by mixing recombinant SpCas9 with sgRNA at a 1:2 molar ratio. Chemically synthesized sgRNAs (Synthego) carried 2′-O-methyl and 3′-phosphorothioate modifications at the first and last three nucleotides; IVT sgRNAs were assembled at matched molar doses. In DLB-1, RNPs for the two guides were prepared separately and mixed 1:1 immediately before electroporation to co-target the two adjacent cut sites. Single-guide electroporations were not pursued in this comparative study.

### 4.5. Lipofection

Two different lipid-based transfection strategies were employed to introduce the CRISPR-Cas9 complex into cells:

Lipid nanoparticles (LNPs) from Diversa Technologies were used for the encapsulation and delivery of CRISPR-Cas9 complexes in DLB-1 cells. The LNPs were fluorescently labeled with FLUOGREEN (Ex/Em = 495/503 nm) to enable visualization and uptake analysis by fluorescence and confocal microscopy. Formulations were prepared according to the manufacturer’s instructions (Ref. DIV063F1), involving ethanol injection of sgRNA into citrate buffer (pH 3), followed by buffer exchange into HEPES buffer (pH 7.4) using Amicon Ultra 10 kDa centrifugal filters. Cas9 protein (20 µg in 50 µL HEPES) was gently mixed with the sgRNA-loaded LNPs and incubated for 4 h at room temperature, then stored overnight at 4 °C. DLB-1 cells were seeded in 24-well plates at 70–80% confluency the day before transfection. The prepared LNP formulation was added directly to the culture medium without additional reagents. Both in vitro transcribed (IVT) sgRNAs and chemically modified sgRNAs (Synthego) were used for formulation and delivery. After 24–48 h of incubation at 25 °C, cells were imaged to evaluate nanoparticle uptake and subsequently subcultured for 7 days before genomic DNA extraction and mutation analysis. All procedures were carried out under RNase-free conditions, avoiding surfactants such as SDS, Triton X-100, or Tween-20 to preserve nanoparticle stability.

### 4.6. Mutation Detection and Analysis

Following transfection, genomic DNA was extracted from cells using one of three protocols: (i) QuickExtract™ DNA Extraction Solution (Lucigen), (ii) Chelex^®^ 100 Resin (Bio-Rad), and (iii) the DNeasy Blood & Tissue Kit (Qiagen). For the QuickExtract protocol, cells were lysed directly in the solution by heating at 65 °C for 10 min, followed by 95 °C for 5 min to inactivate enzymatic activity, as per manufacturer instructions. For Chelex extraction, cell pellets were resuspended in 100 µL of 10% Chelex solution with 1 µL of Proteinase K, incubated at 56 °C for 15 min, and then at 100 °C for 1 h. In the DNeasy protocol, DNA was purified according to the manufacturer’s guidelines using silica-membrane spin columns.

Each extracted DNA sample was used as a template for PCR amplification of the *ifi27l2a* target region. Initial amplifications were conducted using AmpliTaq Gold DNA Polymerase (Applied Biosystems), following the manufacturer’s protocol. However, this approach yielded inconsistent results across extraction methods, including weak or nonspecific amplification.

To compare the performance of different polymerases and DNA templates, we tested three commercial Taq-based enzymes—MyTaq™ Red Mix (Bioline), AmpliTaq Gold (Applied Biosystems), and GoTaq^®^ Flexi (Promega)—on three target regions (*ifi27l2a*-P1, P2, and PE), using genomic DNA obtained with the three extraction methods above, as well as crude seabass DNA as a control. PCR reactions were carried out according to each manufacturer’s instructions and resolved on 1% agarose gels stained with SYBR™ Safe (Invitrogen, Waltham, MA, USA). As shown in Supplementary Figure S5, amplification quality varied depending on the enzyme-template combination. In general, DNA extracted with Chelex and the DNeasy Kit yielded stronger and more specific bands, whereas QuickExtract often resulted in weaker or diffuse products, particularly when combined with Taq-based enzymes. Among the polymerases tested, the Bioline enzyme showed the broadest compatibility across conditions, but none matched the consistency obtained with a high-fidelity enzyme.

To improve specificity and robustness, we subsequently employed Platinum™ SuperFi™ II DNA Polymerase (Thermo Fisher Scientific), a proofreading enzyme optimized for difficult or GC-rich templates. Using this polymerase, consistent and high-quality amplification was achieved across all extraction methods, including QuickExtract, as illustrated in Supplementary Figure S6. These reactions were performed under standard conditions recommended by the manufacturer, and products were resolved on 1% agarose gels stained with SYBR™ Safe.

To assess genome editing efficiency, PCR products were denatured at 95 °C for 5 min and slowly cooled to room temperature to promote reannealing and heteroduplex formation. The resulting DNA mixtures were loaded onto 10% non-denaturing polyacrylamide-agarose gels, stained with SYBR™ Safe, and run at 120 V for 20 min. The presence of heteroduplex bands was interpreted as evidence of CRISPR-induced indels and used to estimate editing efficiency. To validate and further characterize these editing events, PCR products were purified and subjected to Sanger sequencing using the BigDye Terminator v3.1 Cycle Sequencing Kit (Applied Biosystems), followed by capillary electrophoresis on an ABI 3500xl DNA Analyzer. The resulting chromatograms (.ab1 files) were analyzed with the ICE (Inference of CRISPR Edits) tool, which quantified indel frequencies and identified specific insertions or deletions at the target locus, thus providing both qualitative and quantitative confirmation of genome editing outcomes.

### 4.7. Fluorescence Microscopy

Fluorescence microscopy was performed to evaluate transfection efficiency and intracellular localization of delivered components. To visualize the intracellular distribution of Cas9, Cy3-labeled Cas9 protein (1.5 µg/µL) and Cy5-labeled Cas9 protein (9 µg/µL), both acquired through the Proteomics Service of the Andalusian Centre for Developmental Biology (CABD, a joint institute of Pablo de Olavide University and the Spanish National Research Council, Seville, Spain), were used in the transfection complexes. This dual labeling strategy enabled real-time tracking of protein uptake and subcellular distribution. Fluorescence signals were captured using the AZ100 wide-field fluorescence microscope, with optimized exposure settings to minimize photobleaching. The intensity and localization patterns of Cas9-Cy3 and Cas9-Cy5 were analyzed using Fiji (ImageJ), version 2.16.0/1.54p. Rhodamine123 staining was applied as a cytoplasmic counterstain to highlight mitochondria in electroporated cells. This stain was omitted in lipofection experiments to avoid overlap with the green fluorescence of FLUOGREEN-labeled lipid nanoparticles.

Two imaging techniques were used:

Widefield fluorescence microscopy (AZ100, Nikon, Tokyo, Japan): This system was employed to visualize transfected cells, particularly those electropored and transfected with Diversa LNPs. Due to filter limitations, only Cy3-labeled Cas9 fluorescence was detectable, while Cy5 fluorescence remained outside the detection range. The fluorescent signal of FLUOGREEN-labeled Diversa LNPs was successfully captured, allowing the assessment of nanoparticle uptake and intracellular distribution.

Confocal laser scanning microscopy (Leica TCS SPE, Berlin, Germany): High-resolution confocal imaging was performed to visualize the intracellular distribution of Cy5-labeled Cas9 and FLUOGREEN-labeled Diversa LNPs, providing detailed insights into the uptake and subcellular localization of lipofected components. Cells were cultured in 24-well BioLite™ Microwell Plates (Thermo Fisher Scientific) and imaged using a Leica TCS SPE laser scanning inverted confocal microscope equipped with a 5× objective lens (HC PL S-APO 5×/0.15 DRY). This system includes laser lines at 488 nm and 635 nm for selective excitation of FLUOGREEN and Cy5 fluorophores, respectively. Emission was detected using wide bandpass filters set at 500–600 nm (green) and 645–730 nm (red), with channels acquired sequentially to prevent spectral overlap. Photomultiplier gain and laser power were adjusted to avoid pixel saturation, and the pinhole was set to 1.0 Airy unit. Images were acquired at a resolution of 1024 × 1024 pixels. For volumetric analysis, z-stack series spanning 100–150 μm in depth were captured and processed using FIJI-ImageJ to generate three-dimensional projections and videos.

Newly, high-resolution confocal imaging was conducted to track Cy5-labeled Cas9 electroporated cells. Leica TCS SPE laser scanning inverted confocal microscope equipped with a 63x(HC PL APO CS2 63x/1.40 OIL UV) objective lens was used to conduct experiments. μ-Slide 8 well high glass bottom wells from Ibidi with 170 ± 5 μm bottom were used to obtain optimal resolution images. This microscope is equipped with several laser lines, enabling different wavelengths (488 nm and 635 nm) to be excited to reveal Rhodamine 123 (Sigma Aldrich R8004, Burlington, MA, USA) and Cy5, respectively. Green and red fluorescence emission was collected using 500–600 and 645–730 wide emission slits, respectively, in sequential mode to avoid any interference between channels. In addition, photomultipliers and laser excitation sensitivity was set to prevent pixel saturation, the pinhole was set to 1.0 Airy unit and images of 1024 × 1024 pixels were acquired. Finally, images and videos from z-stacks were generated using FIJI-ImageJ software.

### 4.8. Fluorescence Correlation Spectroscopy

Fluorescence correlation spectroscopy (FCS) was used to study the Cas9 protein under the conditions of two of the different delivery methods (specified in the main text). For the electroporation conditions Cas9 labeled with Cy3 was used, while for the lipofection with Diversa LNPs a Cy5 label was utilized, due to limitations in reagent availability. The equipment used in the FCS measurements was provided with different components to excite the Cy3 or Cy5 dyes. For the Cy3, the light of a CW diode laser at 532 nm (Oxxius, LCX-532S, FR, Lannion, France) was coupled to a monomode optical fiber (Oxxius, PM460-HP, FR, Lannion, France), collimated (Schäfter & Kirchhof, 60FC-4-A6.2-01-DI, DE, Hamburg, Germany) and redirected by a dichroic mirror (Semrock, Brightline BS R532, Rochester, NY, USA) and focused into the sample by a microscope objective (Olympus, UPLSAPO 60xW/1.20, water immersion, Tokyo, Japan) mounted in an inverted microscope (Olympus, IX71). The resulting fluorescence was collected by the same objective, directed through a pinhole (Thorlabs, Ø = 150 μm, Newton, NJ, USA) and split into two beams by a nonpolarizing beamsplitter cube (Newport, 05BC17 MB.1, Newton, NJ, USA). The fluorescence was separated from scattered light through a band-pass filter (Semrock, Brightline HC 582/75, USA). Each of these beams was then focused onto an avalanche photodiode (MPD50CTC APD, Ø = 50 μm, MPD, IT). For the Cy5, a different setup was used, the laser was changed to a CW diode laser at 635 nm (Oxxius, LBX-638, FR), the optical fiber (Thorlabs, RB41A1, Newton, NJ, USA), the collimator (Schäfter & Kirchhof, 60FC-4-A7.5-01-DI, DE, Hamburg, Germany), the dichroic mirror (Semrock, Brightline BS R635, Rochester, NY, USA), the pinhole (Thorlabs, Ø = 100 μm, Newton, NJ, USA) and band-pass filter (Semrock, Brightline HC 679/41, Rochester, NY, USA) were changed to match the excitation and emission of the dye, as well as the focal volume. The signals from the detectors were processed and recorded by two TCSPC-modules (SPC 132, Becker & Hickl GmbH, Berlin, DE, Germany) for the Cy3 and a module (MultiHarp 150, PicoQuant, DE, Berlin, Germany) for the Cy5 measurements.

A 100 μL drop of each sample was deposited on clear flat bottom black polystyrene 96-well microplate. The measurements were collected at 25.0 ± 0.5 °C. An excitation laser power of P = 50 μW was chosen for the Cy3 measurements, while a power of P = 200 μW was selected for the Cy5, corresponding to a mean irradiance of $I_{0}/2$ = (0.035 ± 0.003) kW cm^−2^ and $I_{0}/2$ = (0.33 ± 0.01) kW cm^−2^, respectively. Even though a power series could not be performed to select the excitation power, photobleaching is unlikely due to low irradiances. Currently, an aggregation study on the Cas9 protein is underway to assess these issues.

The curves were obtained from the binary data using a custom routine that runs under Python 3.10 using Peulen’s library [39] and analyzed using a fit function with two diffusion correlation times and a triplet term:

(1) $$G\left(t\right)=\frac{1}{N}\left[{R\left(1+\frac{t}{t_{D,1}}\right)}^{-1}{\left(1+\frac{t}{\omega^{2}t_{D,1}}\right)}^{-\frac{1}{2}}+{\left(1-R\right)\left(1+\frac{t}{t_{D,2}}\right)}^{-1}{\left(1+\frac{t}{\omega^{2}t_{D,2}}\right)}^{-\frac{1}{2}}\right](1+A_{T}e^{\frac{-t}{t_{T}}})+b_{0}$$

where t is the correlation time, N is the number of molecules in the focal volume, $t_{D,1}$ and $t_{D,2}$ are the diffusion correlation time of the species diffusing through the focal volume weighted by R, ω is the aspect ratio of the focal volume, $A_{T}$ is the amplitude of the triplet term with triplet lifetime $t_{T}$, and $b_{0}$ is the baseline value for the correlation curve. Two diffusion terms had to be used due to the presence of the free label dye in solution.

To calibrate the sample volume, Rhodamine 6G and Cy5 were used as reference with diffusion coefficient values of (4.3 ± 0.3) × 10^−10^ m^2^ s^−1^ and of (3.6 ± 0.1) × 10^−10^ m^2^ s^−1^, respectively, at 25 °C, obtained after revision of the literature values [40,41,42,43]. For the measurements with Cy3, we obtained with Rhodamine 6G a mean diffusion correlation time of (525 ± 4) μs giving a mean radial 1/e^2^ radius of $W_{xy}$ = (0.95 ± 0.03) μm and a mean focal volume of V = (16 ± 2) μm^3^; for the case of the measurements with Cy5, a mean diffusion correlation time of (271 ± 4) μs was obtained, with a $W_{xy}$ = (0.62 ± 0.01) μm and a mean focal volume of V = (3.8 ± 0.3) μm^3^, using the following equation:

(2) $$D=\frac{{W_{xy}}^{2}}{4t_{D}}$$

Diffusion coefficients were determined using the Stokes-Einstein:

(3) $$D=\frac{k_{B}T}{6\pi \eta R_{H}}$$

where $k_{B}$ is Boltzmann’s constant, T is the temperature, η is the dynamic viscosity of the solvent, and $R_{H}$ is thehydrodynamic radius of the particle. Values for the viscosity of water were set at η (25 °C) = 0.89 cP.

### 4.9. Flow Cytometry

To evaluate the efficiency of electroporation-mediated delivery of Cas9-Cy5, we used SaB-1 and DLB-1 marine fish cell lines. Cells were seeded in 24-well plates and allowed to reach approximately 90% confluence. On the day of electroporation, cells were detached using 0.25% Trypsin-EDTA, counted, and 100,000 cells per condition were collected. After washing with PBS, cells were resuspended in buffer R at a concentration of 1 × 10^7^ cells/mL and incubated with Cas9 protein conjugated to Cy5 at final concentrations of 1, 2, 3, or 5 µM.

Electroporation was carried out using the Neon NxT Electroporation System (Invitrogen) with three pulse settings: 1400 V 20 ms 1 pulse, 1400 V 20 ms 2 pulses, and 1650 V 15 ms 2 pulses. Non-electroporated controls and mock-electroporated controls (0 µM Cas9-Cy5) were included in the experimental design to control for background fluorescence and treatment effects. Electroporated cells were immediately transferred to L-15 medium supplemented with 10% heat-inactivated FBS and seeded in 24-well plates. Each condition was analyzed in duplicate (n = 2).

Forty-eight hours after electroporation, cells were dissociated and resuspended in 100 µL of flow cytometry buffer (PBS + 1% hiFBS). We acquired 10,000 events per sample on a CytoFLEX flow cytometer (Beckman Coulter, Brea, CA, USA), equipped with a red laser (638 nm) and a 660/10 APC filter to detect the Cy5 signal.

The gating strategy included an initial selection of the cell population on the FSC-A vs. SSC-A plot to exclude debris, followed by a singlet gate on the FSC-A vs. FSC-H plot to eliminate aggregates. The percentage of cells containing Cas9-Cy5 was then determined on an APC-H histogram or APC-H vs. Count plot. The fluorescence threshold for positivity was established using the 0 µM Cas9-Cy5 controls and remained consistent across all samples.

Phase-contrast images were acquired using an inverted Oxion 2053.PLPH microscope and a CMEX-5 Pro camera (Euromex, Duiven, The Netherlands), and processed with ImageFocus Alpha version 1.3.7.29719 and ImageJ software version 1.54p.

### 4.10. Quantification of Axial Separation (Δz)

The z-offset between LNP (green) and Cas9 (red) was quantified in Fiji/ImageJ (2.16.0/1.54p). The AVI stack was opened, channels were split (Image ▸ Color ▸ Split Channels) and treated as single-channel z-stacks (C = 1, Z = 121, T = 1). Twelve small rectangular ROIs were placed in regions showing both signals using ROI Manager and applied identically to both channels. For each ROI and channel, *Z*-axis intensity profiles were obtained (Image ▸ Stacks ▸ Plot *Z*-axis Profile) and the peak slice was defined as the slice with the maximum mean intensity. The axial separation per ROI was computed as Δz (slices) = PeakSlice. “List” tables from Fiji were exported and organized into a tidy table with columns Slice, Channel, ROI and Intensity. Summary calculations were performed in R (v4.x) using readxl/dplyr/tidyr/stringr with a custom script that identifies the per-ROI peak slice in each channel and derives Δz, returning per-ROI values and global summary statistics.

### 4.11. Data Visualization and Analysis

Sequence validation and editing analysis were conducted using a combination of Benchling, GraphPad Prism (v9.5.1), and R (v4.3.1). Chromatograms from Sanger sequencing were first uploaded to Benchling (www.benchling.com) to verify base calling accuracy, annotate sgRNA binding sites and predicted cut sites, and confirm the absence of polymorphisms in wild-type controls.

Editing efficiency was then quantified from ICE (Inference of CRISPR Edits) outputs derived from the validated Sanger data. Indel percentages were plotted using GraphPad Prism to compare editing performance across delivery methods, RNP doses, and cell lines. To visualize the diversity and frequency of edited alleles, ICE-generated .json files were processed using a custom R script with the packages jsonlite, tidyverse, ggplot2, and stringr. The script extracted indel variants and generated color-coded alignments, using a consistent base-color scheme: adenine (green), thymine (red), guanine (orange), cytosine (blue), deletions (gray), and cut site markers (black).

Flow cytometry data were analyzed in R to assess transfection efficiency. Raw CytExpert exports were processed using readxl, dplyr, ggplot2, and ggpubr. The percentage of Cy5-positive cells (%_pos) and mean fluorescence intensity (MFI) were extracted from the “Cas9-Cy5 % Total” and “Cas9-Cy5 Mean APC-H” columns, respectively. Missing MFI entries (e.g., “####”) were replaced with a conservative fixed value (100) for plotting consistency. Three types of plots were generated: line plots for %_pos across electroporation conditions and doses, bar plots of MFI values, and bubble plots integrating both metrics (MFI as point size). Statistical analysis was performed using two-way ANOVA followed by Bonferroni post hoc correction (ggpubr::compare_means), with significance set at *p* < 0.05.

### 4.12. Synthesis and Characterization of SPIONs@Gelatin

#### 4.12.1. Materials

Monodisperse gelatin PH3226 was kindly provided by Gelita (Eberbach, Germany). Glutaraldehyde aqueous solution (25%) was purchased from Acros Organics (Geel, Belgium), and genipin was obtained from Fujifilm Wako (Osaka, Japan). Hydrochloric acid solution (37%) was acquired from Fisher Scientific (Waltham, MA, USA), and sodium hydroxide from Merck (Darmstadt, Germany). Reagent-grade acetone and absolute ethanol were purchased from Scharlau (Spain). Iron(III) chloride hexahydrate (FeCl_3_·6H_2_O, 99%), iron(II) sulfate heptahydrate (FeSO_4_·7H_2_O, 99%), sodium citrate dihydrate (C_6_H_5_Na_3_O_7_·2H_2_O, 98%), ammonium hydroxide (NH_4_OH, 28%), fluorescein isothiocyanate (FITC, 90%) and phosphate-buffered saline (PBS) were obtained from Merck (Rahway, NJ, USA). Milli-Q^®^ deionized water (Millipore, Burlington, MA, USA) was used in all experiments.

#### 4.12.2. Synthesis of Citrate Coated SPIONs

SPIONs coated with citrate (SPIONs@Citrate) were synthesized via a coprecipitation method [44]. Briefly, 45 mmol of FeCl_3_·6H_2_O and 30 mmol of FeSO_4_·7H_2_O (Fe^3+^/Fe^2+^ molar ratio of 1.5) were dissolved in 100 mL of a 10 mM aqueous HCl solution under mechanical stirring (210 rpm). Once the salts were completely dissolved, the solution was heated to 60 °C. Upon reaching this temperature, 770 mmol of NH_4_OH were added, resulting in an immediate color change from brown to black, indicating the formation of magnetite. After 30 s, 1.11 mmol of sodium citrate were added, and the reaction was allowed to proceed for 1 h under constant stirring.

After completion, the reaction mixture was cooled to room temperature and acidified to pH 5 using a 9% HCl solution, under continuous stirring. The magnetite nanoparticles were then separated from the reaction medium using a neodymium magnet. The resulting SPIONs were washed thoroughly with Milli-Q^®^ water (6×) and finally redispersed in Milli-Q^®^ water.

#### 4.12.3. Synthesis of Fluorescent Gelatin Coated SPIONs

Fluorescent gelatin-based magnetic nanoparticles loaded with SPIONs were synthesized using SPIONs@Citrate as the magnetic core. To ensure homogeneous dispersion, an aqueous suspension of SPIONs@Citrate (3.5 mL, 0.72 mg/mL) was sonicated for 5 min (Branson Sonifier, output 1, 95% duty cycle). Immediately after sonication, an equal volume of a FITC-functionalized gelatin solution (40 mg/mL, containing 20 mg of FITC) was added under constant stirring. Fluorescent magnetic nanoparticle formation was subsequently carried out by the desolvation method, following previously established protocols. In this preparation, magnetic separation was employed for fluorescent SPIONs@Gelatin isolation using an external magnet over 24 h [44]. The supernatant was then removed, and the resulting fluorescent magnetic gelatin nanoparticles were resuspended in 2 mL of PBS.

#### 4.12.4. Bioconjugation of Fluorescent Gelatin-Coated SPIONs with Cas9–sgRNA Complexes

To directly immobilize the two sgRNA complexes (derived from DLB-1 and SaB-1 species) onto the fluorescent SPIONs@Gelatin nanoparticles, the method described by Puertas was modified accordingly [45]. For this purpose, 200 μL of MES buffer solution (50 mM) containing EDC (0.0432 M) and Sulfo-NHS (0.0648 M) was added to 5 mg of fluorescent SPIONs@Gelatin dispersed in 800 μL of MES buffer (50 mM), yielding a final reaction volume of 1 mL. The mixture was homogenized using a vortex mixer and then incubated at 37 °C for 30 min under orbital shaking (200 rpm). Following activation, the supernatant was removed by pipetting after centrifugation at 13,000 rpm for 15 min, and the activated nanoparticles were resuspended in 1 mL of Milli-Q water using a 2-min ultrasonic bath. This washing step was repeated twice.

Next, 100 μg of the desired sgRNA complexes were added to the activated fluorescent SPIONs@Gelatin suspension in Milli-Q water, and the mixture was incubated at 37 °C under orbital shaking (200 rpm) for 16 h. After conjugation, the fluorescent SPIONs@Gelatin-RNPs were collected by centrifugation (13,000 rpm, 15 min, 4 °C), and the supernatant was discarded. The pellet was resuspended in 1 mL of Milli-Q water and sonicated for 2 min. This washing step was repeated two additional times.

Finally, the fluorescent SPIONs@Gelatin-RNPs were resuspended in sterile-grade water for further use.

### 4.13. Physicochemical and Biological Characterizations of SPIONs@Gelatin and SPIONs@Gelatine-RNP

#### 4.13.1. Structural Characterization

The crystalline phase of the SPIONs@Gelatin was characterized by X-ray diffraction (XRD) using powder sample and a Philips PW1710 diffractometer (Panalytical, Brighton, UK) equipped with a Cu Kα radiation source (λ = 1.54186 Å). Data were collected over a 2θ range of 10° to 80°, with a step size of 0.02° and a counting time of 10 s per step. The XRD pattern of SPIONs@Gelatin is presented in Supplementary Figure S8A. The observed diffraction peaks correspond to the characteristic reflections at (220), (311), (400), (422), (511), (440), (620), and (553), in agreement with the standard pattern from the Joint Committee on Powder Diffraction Standards (JCPDS card no. 19-0629), which is consistent with the inverse spinel crystalline structure of magnetite [46]. In addition, a broad band observed at around 20° in the pattern is indicative of the amorphous nature of the gelatin coating [47].

#### 4.13.2. Morphological Characterization

The morphology and size of SPIONs@Gelatin nanoparticles were analyzed by scanning transmission electron microscopy (STEM) using a Zeiss Gemini 450 Field Emission Scanning Electron Microscope (Carl Zeiss, Oberkochen, Germany). Particle size distribution was determined by measuring 400 individual nanoparticles using ImageJ software. As shown in Supplementary Figure S8B(a), the SPIONs@Gelatin exhibit a relatively uniform distribution of spherical core–shell structures. The darker central regions correspond to the superparamagnetic iron oxide cores (SPIONs), while the lighter halos are indicative of the gelatin coating. The particles display good dispersion and minimal aggregation, suggesting effective surface functionalization with gelatin. The corresponding size distribution histogram (Supplementary Figure S8B(b)) shows an average particle diameter of approximately 100 nm, with a narrow size range, indicating uniformity in synthesis.

Upon jugation with the Cas9–sgRNA complex, a morphological change is observed. Both bright-field (Supplementary Figure S8B(c)) and dark-field (Supplementary Figure S8B(d)) STEM images reveal that while the spherical morphology of the nanoparticles is preserved, there is a clear tendency toward aggregation. This behavior is likely due to intermolecular interactions between the gelatin-coated surfaces and the biomolecular components of the Cas9–sgRNA complex.

#### 4.13.3. Surface Characterization

The FT-IR spectra of gelatin, SPIONs@Citrate, and SPIONs@Gelatin are presented in Supplementary Figure S8C. The spectrum of pure gelatin exhibits characteristic absorption bands at 3301 cm^−1^ (ν_N–H_), 1672 cm^−1^ (ν_C = O_, amide I), and 1473 cm^−1^ (δ_N–H_, amide II), which are consistent with its polypeptide structure and indicative of the presence of amide functional group [48,49]. In the spectrum of SPIONs@Citrate, several distinct absorption bands are observed at 3348, 2923, 2862, 1568, 1373, 1367, 1356, and 1063–1068 cm^−1^. These signals correspond to O–H stretching (3348 cm^−1^), C–H stretching of methylene groups (2923 and 2862 cm^−1^), asymmetric and symmetric stretching vibrations of carboxylate groups (1568, 1373, 1367, and 1356 cm^−1^), and C–O or C–C stretching modes (1063–1068 cm^−1^). The presence of these bands confirms the successful functionalization of the SPIONs surface with citrate molecules, which act as stabilizing and anchoring agents for subsequent biopolymer coating [50]. The spectrum of SPIONs@Gelatin retains the characteristic bands of both gelatin and citrate, demonstrating the successful encapsulation of citrate-functionalized SPIONs within the gelatin matrix. In addition, a prominent absorption band at 580 cm^−1^ is observed, corresponding to the Fe–O stretching vibration, which confirms the presence of magnetite as the magnetic core of the nanostructure [51]. The coexistence of organic (gelatin and citrate) and inorganic (Fe_3_O_4_) vibrational modes in the spectrum supports the effective stepwise functionalization of SPIONs, resulting in a stable citrate-modified, gelatin-coated magnetic core–shell nanostructure.

The Cas9–sgRNA quantification procedure was carried out following the manufacturer’s protocol using the Micro BCA Protein Assay Kit (Thermo Fisher Scientific). Bovine serum albumin (BSA) was used as the protein standard to construct the calibration curve. Standard BSA solutions were prepared at concentrations of 0, 3.125, 6.25, 12.5, 25, 50, 100, and 200 µg/mL (Supplementary Figure S8C(b)). Each standard and nanoparticle-protein sample was measured in triplicate in a 96-well microplate by adding 100 µL per well. After incubation, the absorbance of each well was measured at 562 nm using a microplate reader. The absorbance values obtained from the BSA standards were used to construct a linear calibration curve, resulting in the following regression equation:

y = 0.0092x ± 2.44  ×  10^−4^ (R^2^ = 0.998) (4)

where y corresponds to the absorbance at 562 nm and x to the protein concentration in µg/mL. This linear relationship allowed accurate determination of the concentration of Cas9–sgRNA conjugated to the SPIONs@Gelatin nanoparticles based on the measured absorbance values. To evaluate the efficiency of different Cas9–sgRNA complexes upon immobilization, it tested two constructs, DLB-1 and SaB-1, starting from 100 µg of each complex. The immobilized DLB-1 complex exhibited a cleavage efficiency of 42.90%, corresponding to 42.90 µg retained on the nanoparticle surface. Similarly, the SaB-1 complex reached an efficiency of 38.68%, equivalent to 38.68 µg immobilized. These results indicate that both complexes retain a significant proportion of their mass upon immobilization, with DLB-1 showing slightly higher efficiency under the tested conditions. The remaining fraction, not bound to the Fluorescent SPIONs@Gelatin, accounted for 57.10 µg and 61.32 µg for DLB-1 and SaB-1, respectively.

#### 4.13.4. DLS Characterization

Hydrodynamic size and ζ-potential of fluorescent SPIONs@Gelatin and SPIONs@Gelatin-RNPs were measured using a Zetasizer Nano ZS (Malvern Instruments, Worcestershire, UK) equipped with a 633 nm He–Ne laser at a 173° scattering angle and room temperature. Measurements were conducted in Milli-Q water at ~0.4 mg/mL magnetic nanomaterial concentration and pH 6.5. In Table 2, the hydrodynamic diameter, PDI, and ζ-potential values of SPIONs@Gelatin and SPIONs@Gelatin-RNPs are presented. SPIONs@Gelatin exhibited a relatively small size (112.4 ± 44.0 nm) with a low polydispersity index (0.11), indicating a homogeneous population and good colloidal stability in aqueous medium. Upon bioconjugation, a significant increase in hydrodynamic diameter was observed (629.5 ± 180.7 nm), which is consistent with the formation of larger conjugates or mild aggregation induced by the biomolecule binding. Despite this increase in size, the PDI remained low (0.17), suggesting that the dispersion maintained acceptable uniformity. The ζ-potential also became more negative after bioconjugation (–25.0 ± 6.1 mV vs. –13.3 ± 3.6 mV), likely due to the presence of negatively charged functional groups on the biomolecules, which may contribute to enhanced electrostatic stabilization.

#### 4.13.5. Optical Characterization

Optical characterization of fluorescent SPIONs@Gelatin and SPIONs@Gelatin-RNPs was performed using a Leica TCS SP8 SMD confocal laser scanning microscope with multispectral fluorescence imaging capability. Technical parameters included a resolution of 1024 × 1024 pixels, bidirectional *X*-axis scanning, and a HC PL APO CS 63×/1.40 oil immersion objective. Excitation was achieved using the 488 nm and 552 nm laser lines for FITC-labeled SPIONs@Gelatin and Cy5-labeled Cas9, respectively [52].

Confocal microscopy images (Figure 3C) provide fluorescence-based visualization of SPIONs@Gelatin and their bioconjugation with fluorescent Cas9–sgRNA complexes. Figure 3C(a) shows green fluorescence from SPIONs@Gelatin, indicating effective functionalization with the fluorophore. Figure 3C(b) displays red fluorescence corresponding to the labeled Cas9–sgRNA complex. Upon bioconjugation using the DLB-1 complex Figure 3C(c,d), strong colocalization of green and red fluorescence is observed, resulting in a yellow/orange merged signal. This suggests successful and efficient conjugation of Cas9–sgRNA to SPIONs@Gelatin mediated by DLB-1. Similarly, panel d (SaB-1 complex) shows fluorescence colocalization, though to a slightly lower extent compared to DLB-1. These results are consistent with the previously quantified immobilization efficiencies and confirm that both complexes effectively facilitate the attachment of Cas9–sgRNA to the magnetic nanoparticl platform.

#### 4.13.6. Magnetic Characterization

The magnetic properties of the SPIONs@Gelatin nanocomposite were evaluated using a vibrating sample magnetometer (VSM, DMS, Lowell, MA, USA) at room temperature. As shown in Supplementary Figure S8D, the magnetization curve exhibits a typical hysteresis loop with negligible coercivity and remanence, indicating superparamagnetic behavior at room temperature [53]. The saturation magnetization (Ms) was approximately 66 emu/g_Fe3O4_, which confirms the retention of magnetic properties after gelatin coating. The inset of Supplementary Figure S8D displays the zero-field-cooled (ZFC) and field-cooled (FC) magnetization curves recorded under an applied field of 100 Oe measured by a Superconducting Quantum Interference Device (SQUID) Magnetometer (Quantum Design, Darmstadt, Germany). The divergence between the ZFC and FC curves, along with the broad maximum observed in the ZFC curve around the blocking temperature (T_B_ = 90 K), further confirms the presence of superparamagnetic nanoparticles at room temperature [54].

#### 4.13.7. Magnetofection Protocol Using Neodymium-Based Magnetic Plates

Magnetofection was performed using custom magnetic arrays designed to fit under standard 24-well and 96-well tissue culture plates. Each position in the array consisted of a circular neodymium magnet disc (diameter: 10 mm; thickness: 2 mm; strength: N52 grade) placed inside a polypropylene support that aligned precisely beneath each well. The magnetic plate was positioned beneath the culture plate immediately after the addition of SPIONs@Gelatin–RNP complexes.

Cells were seeded at ~20,000 cells/well (96-well format) or ~100,000 cells/well (24-well format) and allowed to adhere overnight at 28 °C. On the day of treatment, SPIONs@Gelatin–RNP complexes were added directly to the culture medium in each well. Immediately after addition, the magnetic array was placed underneath the culture plate to generate a downward magnetic force, enhancing nanoparticle sedimentation and cell contact.

Three volumes of nanoparticle suspension were tested, namely 100, 200, and 500 µL per well, corresponding to final RNP doses of 0.41, 0.82, and 2.05 ng per cell, respectively. Incubations were carried out at 25 °C for 30 or 60 min. After exposure, the culture medium was replaced with fresh L-15 medium supplemented with 10% FBS and 1% penicillin/streptomycin, and cells were cultured for 48 h prior to fluorescence imaging and genomic DNA extraction.

Control conditions lacking magnetic exposure were processed in parallel using identical volumes and incubation times but without the magnetic plate. All experimental steps were conducted in triplicate for each cell line and condition.

## Figures and Tables

**Table 1:** Diffusional properties of the Cas9 at 25 °C under the experimental conditions of two of the delivery strategies: electroporation and lipofection with Diversa LNPs.

Conditions	D/10^−10^ m^2^ s^−1^	$R_{H}$/nm
Electroporation (cell medium)	0.207 ± 0.007	11.9 ± 0.4
Diversa LNPs (HEPES)	0.111 ± 0.008	22 ± 2

**Table 2:** Hydrodynamic diameter (D_H_), polydispersity index (PDI), and ζ-potential of SPIONs@Gelatin and SPIONs@Gelatin-RNPs measured by dynamic light scattering (DLS) in water at pH 6.5. Values are expressed as mean ± standard deviation.

Sample	DH (nm)	PDI	ζ-Potential (mV)
SPIONs@Gelatin	112.4 ± 44.0	0.11	−13.3 ± 3.6
Bioconjugated SPIONs@Gelatin	629.5 ± 180.7	0.17	−25.0 ± 6.1

## Data Availability

The original contributions presented in this study are included in the article/Supplementary Materials. Further inquiries can be directed to the corresponding author(s).

## Supplementary Materials

The following supporting information can be downloaded at https://www.mdpi.com/article/10.3390/ijms262110703/s1.

## Abbreviations

The following abbreviations are used in this manuscript:

CRISPR	Clustered Regularly Interspaced Short Palindromic Repeats
Cas9	CRISPR-associated protein 9
RNP	Ribonucleoprotein complex
sgRNA	Single-guide RNA
LNP	Lipid nanoparticle
SPIONs	Superparamagnetic iron oxide nanoparticles
ICE	Inference of CRISPR Edits
FCS	Fluorescence Correlation Spectroscopy
PCR	Polymerase Chain Reaction
PBS	Phosphate-buffered saline
FBS	Fetal bovine serum
PE P1 P2	Primer pairs used for PCR amplification of *ifi27l2a*
WT	Wild type
KO	Knock-out
DLB-1	Dicentrarchus labrax brain-derived cell line
SaB-1	Sparus aurata brain-derived cell line
DAPI	4′,6-diamidino-2-phenylindole
Cy5	Cyanine 5 (fluorophore)
SEM	Standard error of the mean
GC	Guanine–cytosine content
