# Supplementary material for: Comparative Analysis of CRISPR/Cas9 Delivery Methods in Marine Teleost Cell Lines
Source: Int J Mol Sci. 2025 Nov 3;26(21):10703. doi: 10.3390/ijms262110703 (PMC12610623; doi:10.3390/ijms262110703)
Supplement: Supplementary file 1 [file ijms-26-10703-s001.zip › Supplementary Materials_v2.2.pdf]

Supplementary Materials:

Supplementary Table S1. CRISPOR metrics (with mismatch breakdown)

Species	*guideId*	*MIT spec*	*CFD spec*	*Pred. off-targets*	*Doench'16*	*Azimuth in-vitro*	*Out-of-Frame*	*Lindel*	*Moreno-Mateos*	*Off-targets for 0-1-2-3-4 mismatches + next to PAM*
DLB1-sgRNA1	432forw	46	70	323	45	79	60	68	85	**0–2–11–34–277** 0–2–2–6–2
DLB1-sgRNA2	409forw	38	90	192	59	52	36	67	65	**0–3–4–13–172** 0–0–0–0–1
SaB-sgRNA1	233forw	94	98	18	25	NA	-111	57	62	**0–0–1–2–15** 0–0–0–0–0
SaB-sgRNA2	327forw	90	96	24	51	NA	60	35	78	**0–0–2–4–18** 0–0–1–0–0

Supplementary Table S2. Primers used for amplification of *ifi27l2a* in seabass (DLB-1) and gilthead seabream (SaB-1) cell lines.

Species	Name	Sequence (5′→3′)	Strand	Length (bp)	Tm (°C)	GC (%)	Product (bp)
DLB-1 (seabass)	*ifi27l2a*-PE-F	GGTCAGTGTCGCTGTTCCTT	Plus	20	60,25	55	855
DLB-1 (seabass)	*ifi27l2a*-PE-R	CATCCCACTGCTCCTCCAAC	Minus	20	60,39	60	
DLB-1 (seabass)	*ifi27l2a*-P1-F	TCTTGCTGGTCAGTGTCGC	Plus	19	60,3	57,89	630
DLB-1 (seabass)	*ifi27l2a*-P1-R	TGTTTAAGATCCAGGTCTGCCA	Minus	22	59,36	45,45	
DLB-1 (seabass)	*ifi27l2a*-P2-F	TTACACACACATCTGGCAGACC	Plus	22	60,55	50	419
DLB-1 (seabass)	*ifi27l2a*-P2-R	ATTGCTCTCCCTCAGAACCA	Minus	20	58,34	50	
SaB-1 (gilthead seabream)	*ifi27l2a*-Sa1F	AACAATCGTGTTTTGCCGCC	Plus	20	60,6	50	480
SaB-1 (gilthead seabream)	*ifi27l2a*-Sa1R	GGTAAAGTCAGGCTGGAGAGG	Minus	21	59,79	57,14	
SaB-1 (gilthead seabream)	*ifi27l2a*-Sa2F	TCTTCCCCAAAAATACGGTGC	Plus	21	58,84	47,62	593
SaB-1 (gilthead seabream)	*ifi27l2a*-Sa2R	GCAAACTGGAACAACGTCCAT	Minus	21	59,66	47,62	

Supplementary Table S3. CRISPR/Cas9 sgRNAs targeting *ifi27l2a* in seabass and gilthead seabream.

**Species**	***sgRNA* name**	**Target sequence (genome)**	***sgRNA* oligo (T7)**
Seabass	DLB1-*sgRNA1*	AGGAGTGGCAGCAGGAGGTGTGG	taatacgactcactataGGGAGTGGCAGCAGGAGGTGgttttagagctagaa
Seabass	DLB1-*sgRNA2*	GCTGCTGCGGTTGCTAATGGAGG	taatacgactcactataGGTGCTGCGGTTGCTAATGGgttttagagctagaa
Gilthead seabream	SaB-*sgRNA1*	TGCCGTGGTCGGGGCTCCTTTGG	taatacgactcactataGGCCGTGGTCGGGGCTCCTTgttttagagctagaa
Gilthead seabream	SaB-*sgRNA2*	GCTGCGATTGCTAACGGAGGAGG	taatacgactcactataGGTGCGATTGCTAACGGAGGgttttagagctagaa
All	Universal oligo	—	AAAAGCACCGACTCGGTGCCACTTTTTCAAGTTGATAACGGACTAGCCTTATTTAACTTGCTATttctagctctaaaac

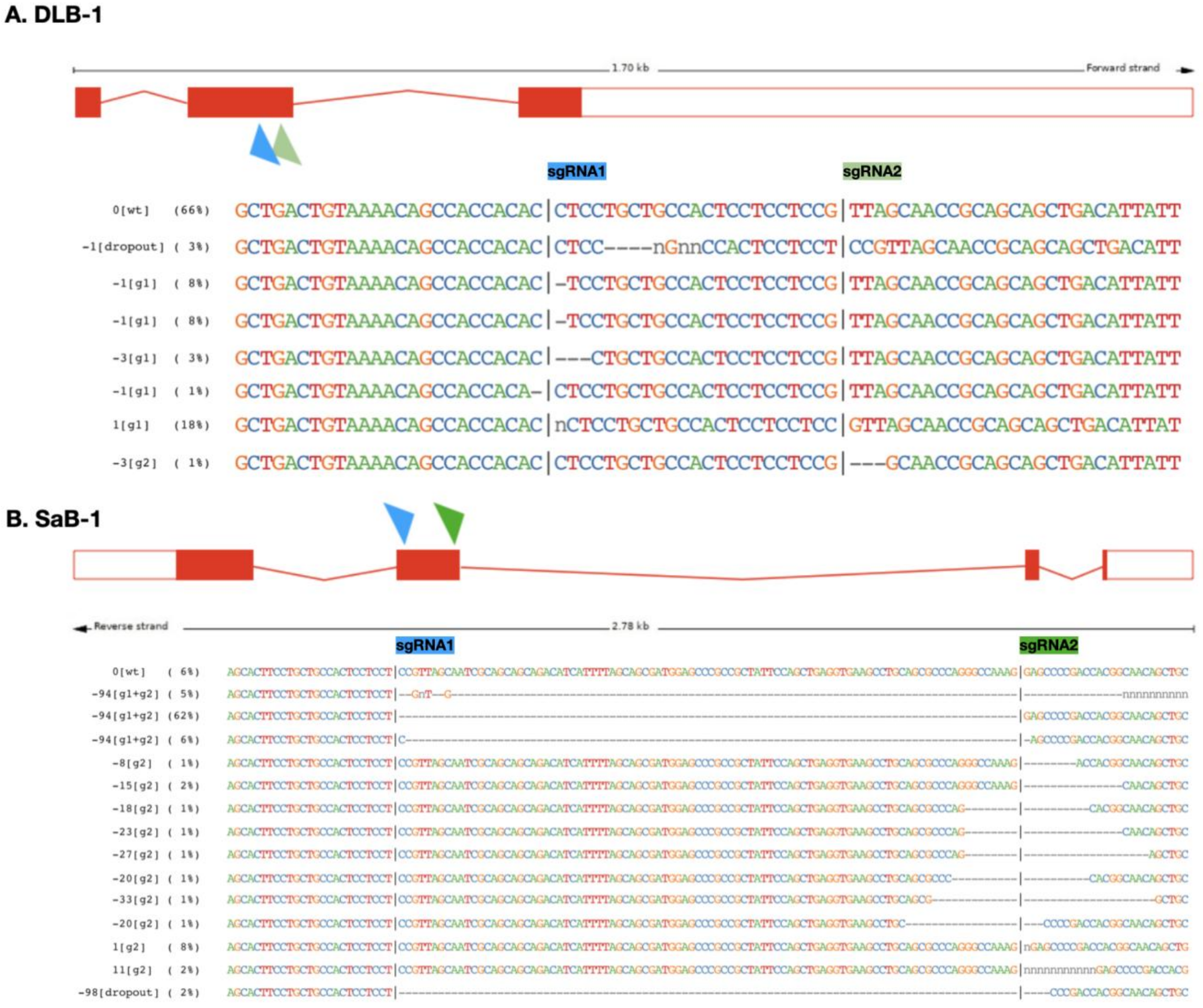

Supplementary Figure S1. ICE analysis of CRISPR/Cas9 editing outcomes at the *ifi27l2a* locus in DLB-1 and SaB-1 cells. A. Results from DLB-1 cells electroporated with Cas9 RNPs targeting *ifi27l2a* using *sgRNA1* (blue triangle) and *sgRNA2* (green triangle). The schematic above represents the genomic structure of the target region, with exons (red boxes) and the relative positions of both guides. Below, the wild-type (wt) sequence and the most frequent indel variants are shown, aligned at the cleavage site. The percentage of each allele corresponds to ICE (Inference of CRISPR Edits) deconvolution results. B. Results from SaB-1 cells edited with the same combination of *sgRNAs*. The genomic organization of the *ifi27l2a* locus is shown above, including exon–intron structure and *sgRNA* target sites. Representative allele sequences are aligned below, showing predominant deletions and insertions around the expected cut sites. The wild-type sequence is shown for comparison. Percentages reflect the relative abundance of each allele as estimated by ICE.

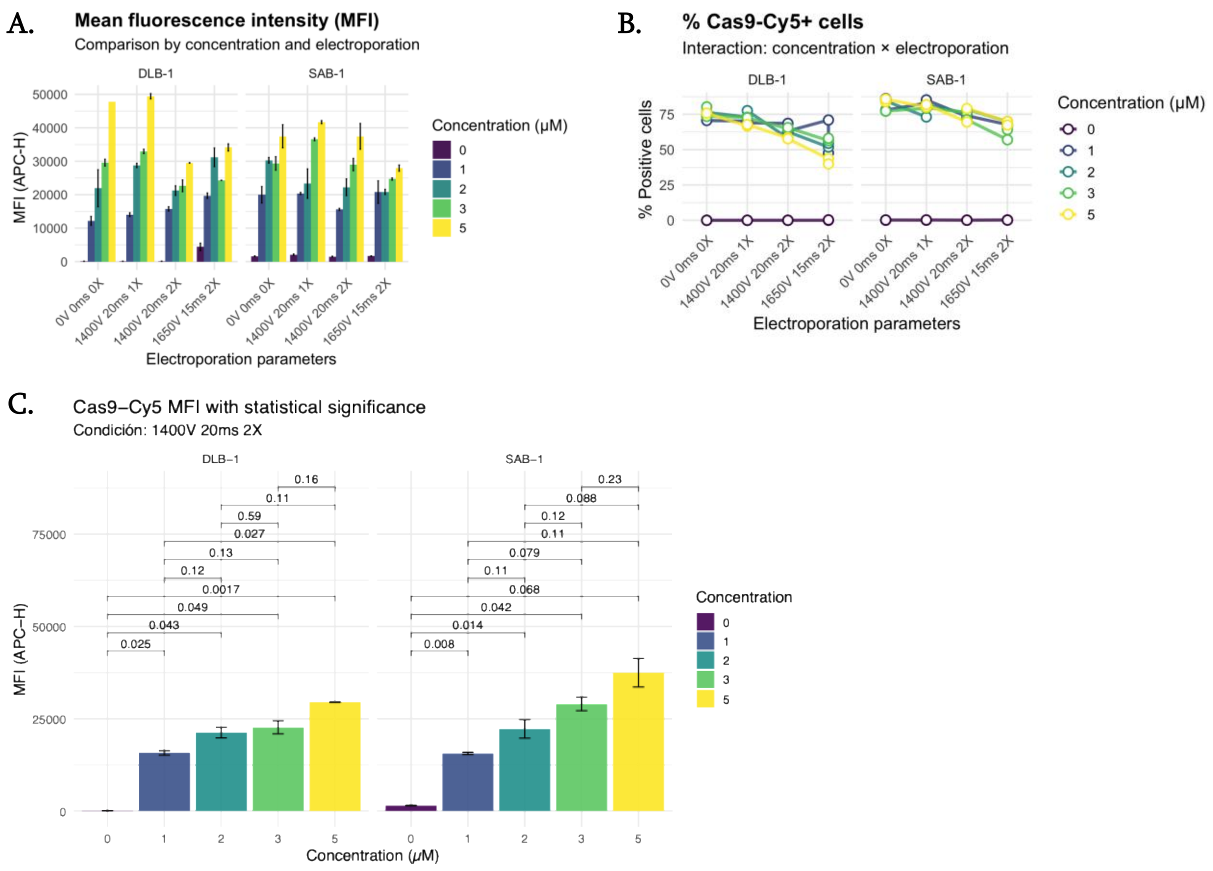

Supplementary Figure S2. Quantitative analysis of Cas9-Cy5 delivery in DLB-1 and SaB-1 cells by flow cytometry. A. Mean fluorescence intensity (MFI) of Cas9-Cy5 (APC-H channel) across electroporation parameters and protein concentrations (0–5 µM) in DLB-1 and SaB-1 cells. Each condition was tested in duplicate, showing a dose-dependent increase in fluorescence, more pronounced under optimized electroporation settings. B. Percentage of Cas9-Cy5–positive cells (% APC+) detected for each condition. While total fluorescence intensity increased with concentration, The proportion of positive cells plateaued at intermediate doses, suggesting a saturation effect. C. MFI values under the 1400 V / 20 ms / 2 pulses condition, highlighting statistical comparisons between concentrations. Bars represent mean ±SD; p-values from 2-way ANOVA with Bonferroni correction are shown. Both cell lines exhibit a significant increase in Cas9 signal with rising concentration, although differences between the highest doses are less pronounced in SaB-1.

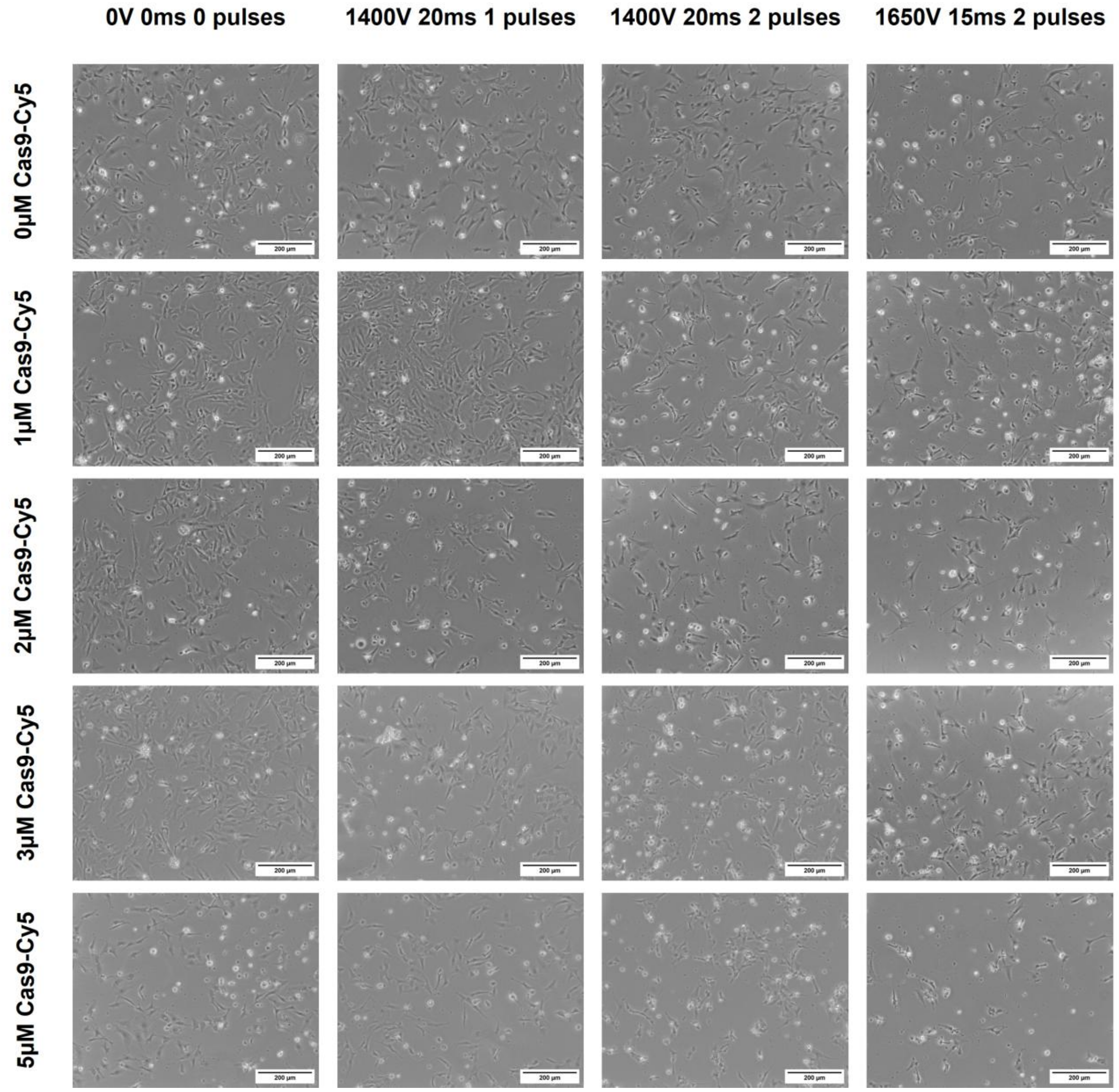

Supplementary Figure S3A. Brightfield images of DLB-1 cells acquired immediately prior to flow cytometry analysis, showing the effects of different electroporation conditions (voltage, pulse number/duration) and Cas9-Cy5 concentrations (0–5 µM). Each row corresponds to a specific Cas9-Cy5 concentration, while each column represents a distinct electroporation setting. Cell morphology and confluency were monitored to assess cytotoxicity and electroporation-related stress. Scale bar = 200 µm.

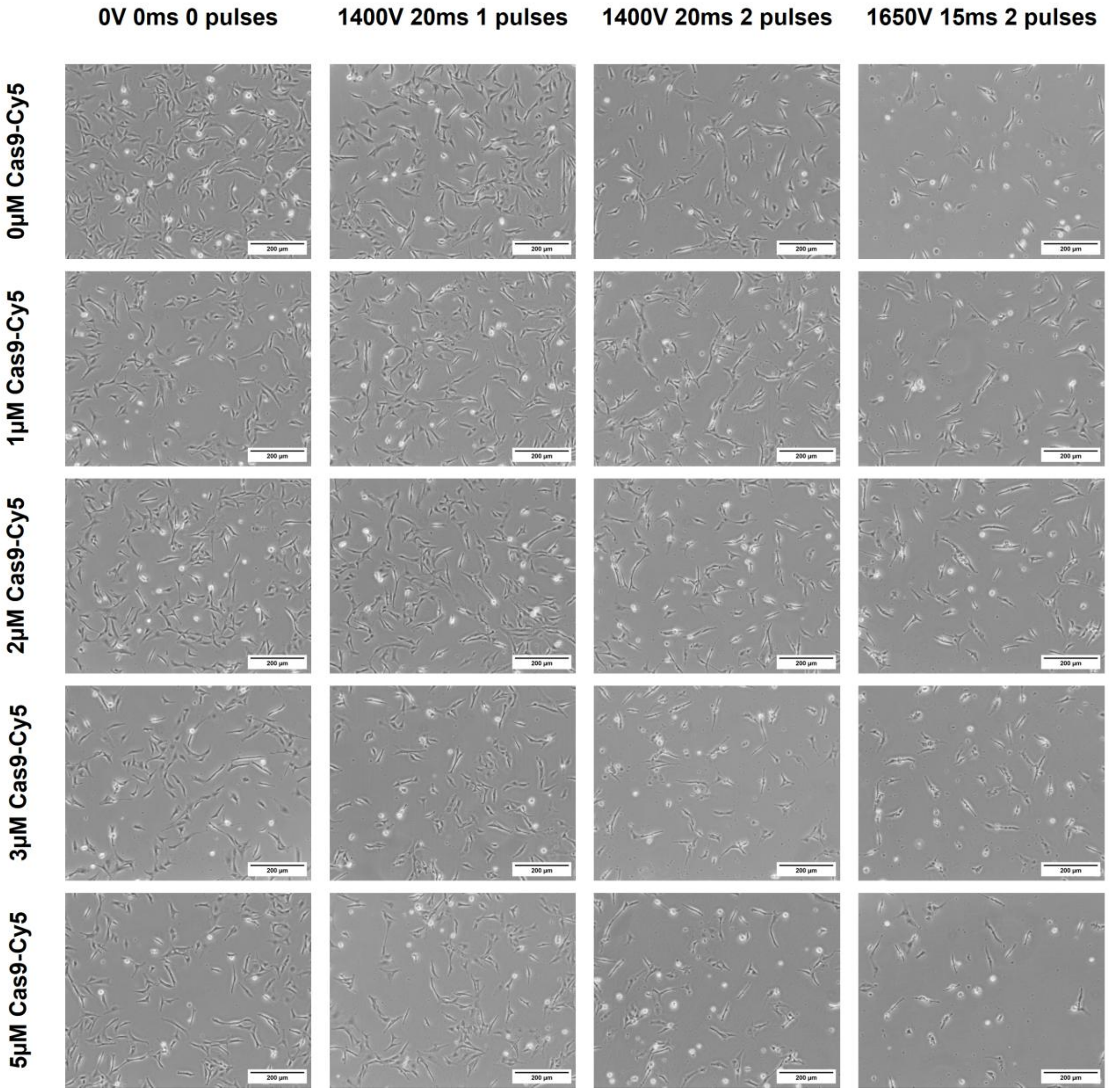

Supplementary Figure S3B. Brightfield images of SaB-1 cells captured immediately before flow cytometry, following identical electroporation protocols as in S5A. Cells were treated with increasing concentrations of Cas9-Cy5 (0–5 µM) and exposed to four electroporation conditions. Visual inspection of cell integrity and density served as a quality control step prior to fluorescence quantification. Scale bar = 200 µm.

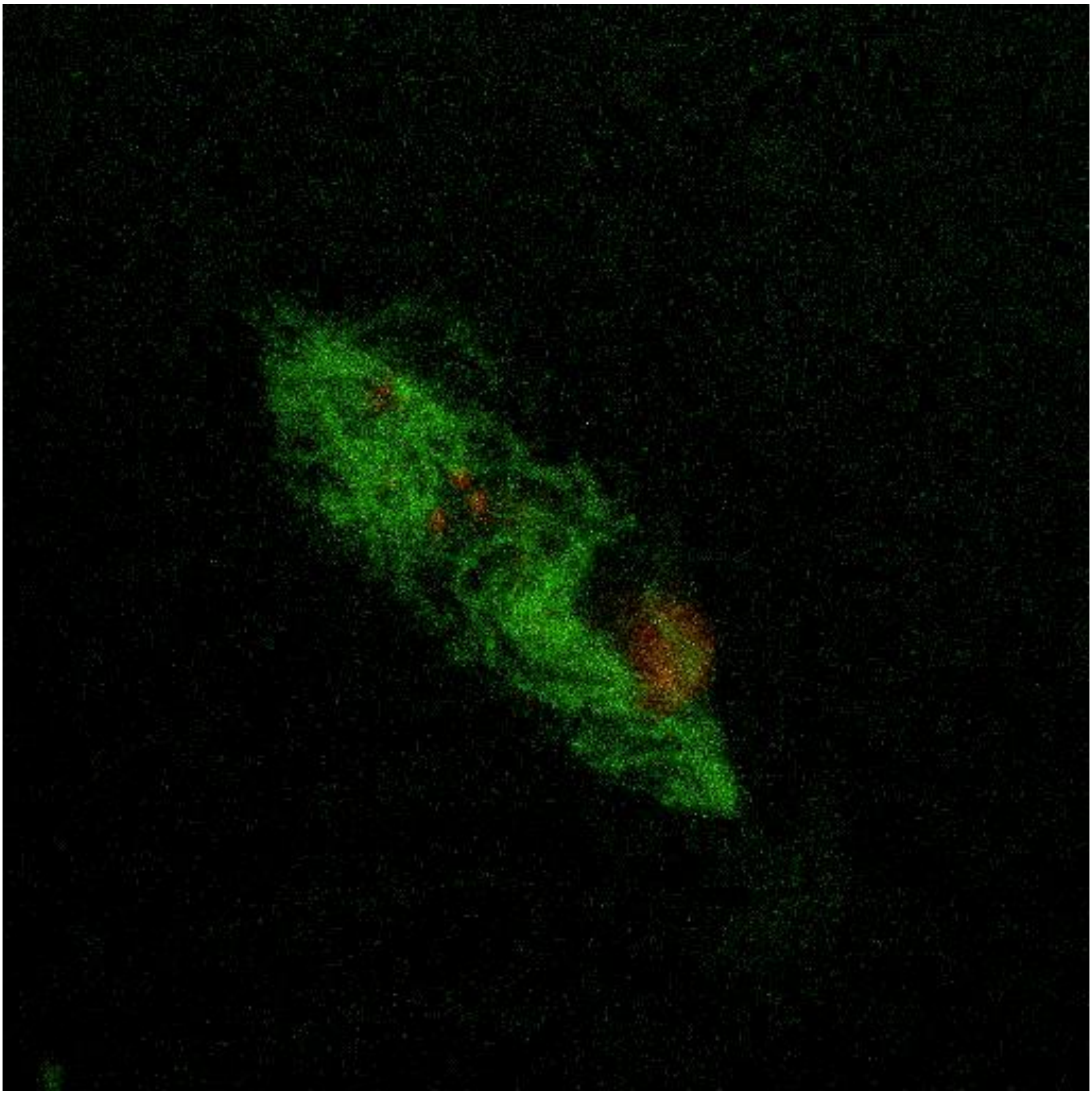

Supplementary Video S1. Intracellular distribution of Cas9-Cy5 following electroporation in DLB-1 cells stained with Rhodamine. Confocal laser-scanning microscopy of a representative DLB-1 cell electroporated with Cas9–Cy5 (red) and stained with Rhodamine123 (green), which labels mitochondria and outlines the cytoplasm. Cas9–Cy5 appears in multiple cytoplasmic foci with perinuclear accumulations; a diffuse, rounded perinuclear region is also visible. Because no nuclear counterstain was used, nuclear localization cannot be confirmed. Retention of Cas9–Cy5 signal at 24 h indicates intracellular persistence, with aggregation and compartmentalization evident. Channels were acquired sequentially.

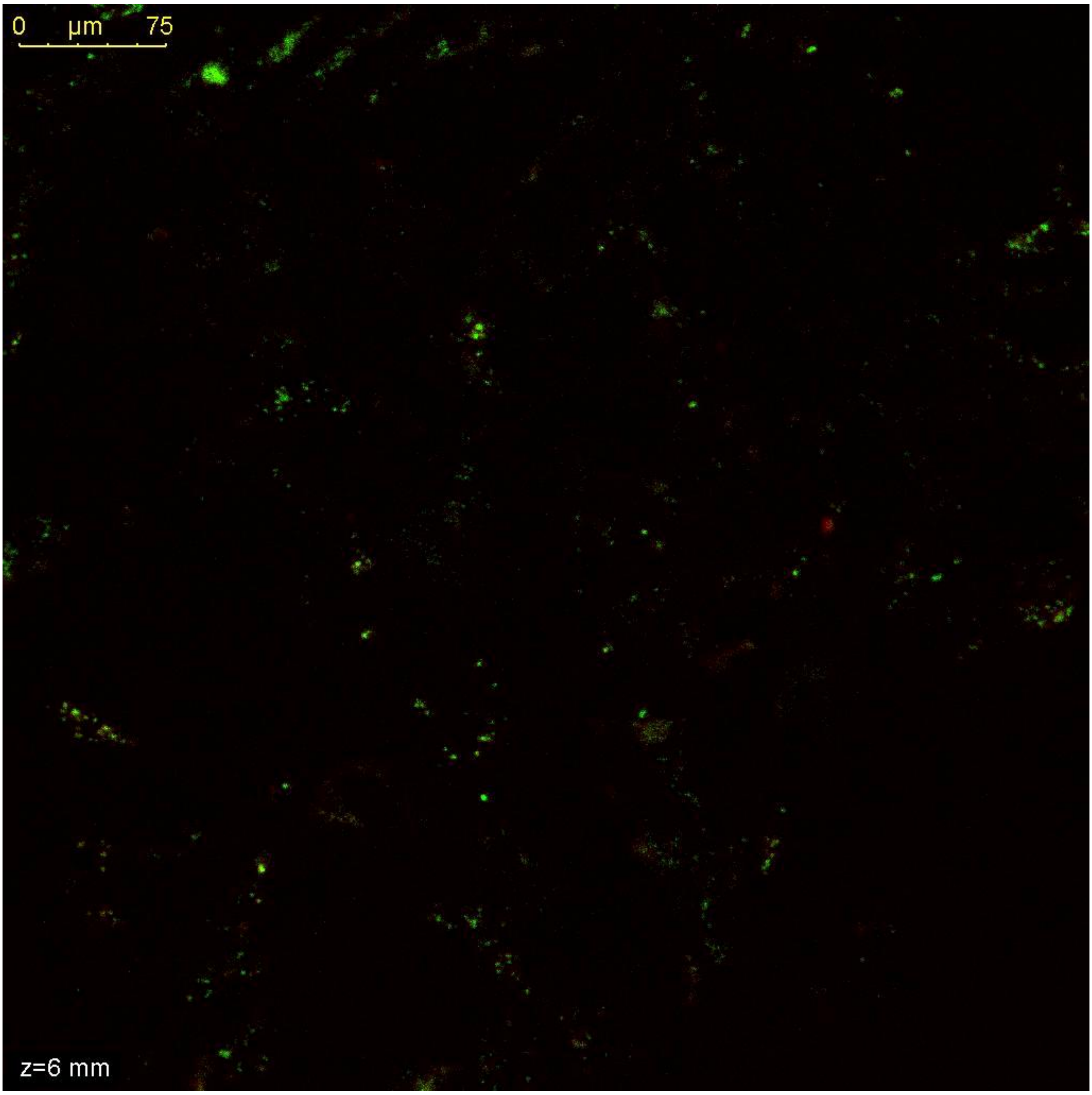

Supplementary Video S2. Confocal imaging showing the subcellular distribution of components following lipofection with Diversa lipid nanoparticles (LNPs). The LNPs (green) predominantly accumulate in upper optical sections, consistent with localization at or near the cell membrane. In contrast, Cas9-Cy5 (red) appears in lower planes, within the cells, concentrated in specific but undefined intracellular regions. This spatial separation suggests a dissociation between nanoparticle retention at the membrane level and Cas9 internalization. Channels were acquired sequentially. No dye-swap or multicolor bead registration was performed; therefore, minor chromatic aberration cannot be excluded. Scale bar = 75 µm

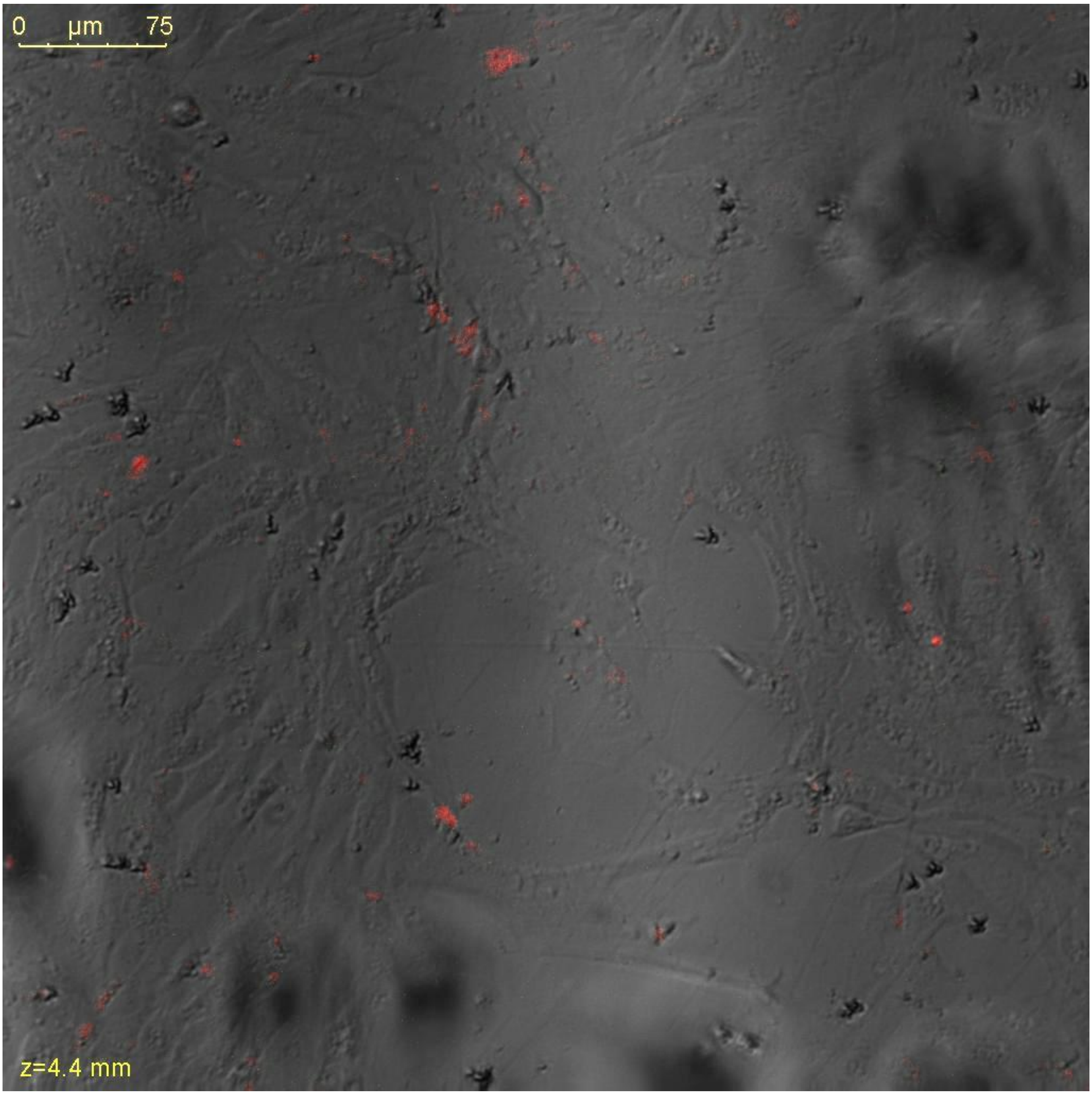

Supplementary Video S3. Imaging of a different region of the cell culture, showing the same distribution pattern: Diversa nanoparticles (green) remain in the upper focal planes, while Cas9-Cy5 (red) is detected in deeper intracellular locations. In this case, a merge with brightfield imaging is included, allowing visualization of cellular morphology and confirming the intracellular localization of Cas9 relative to cell boundaries. Channels were acquired sequentially. No dye-swap or multicolor bead registration was performed; therefore, minor chromatic aberration cannot be excluded. Scale bar = 75 µm.

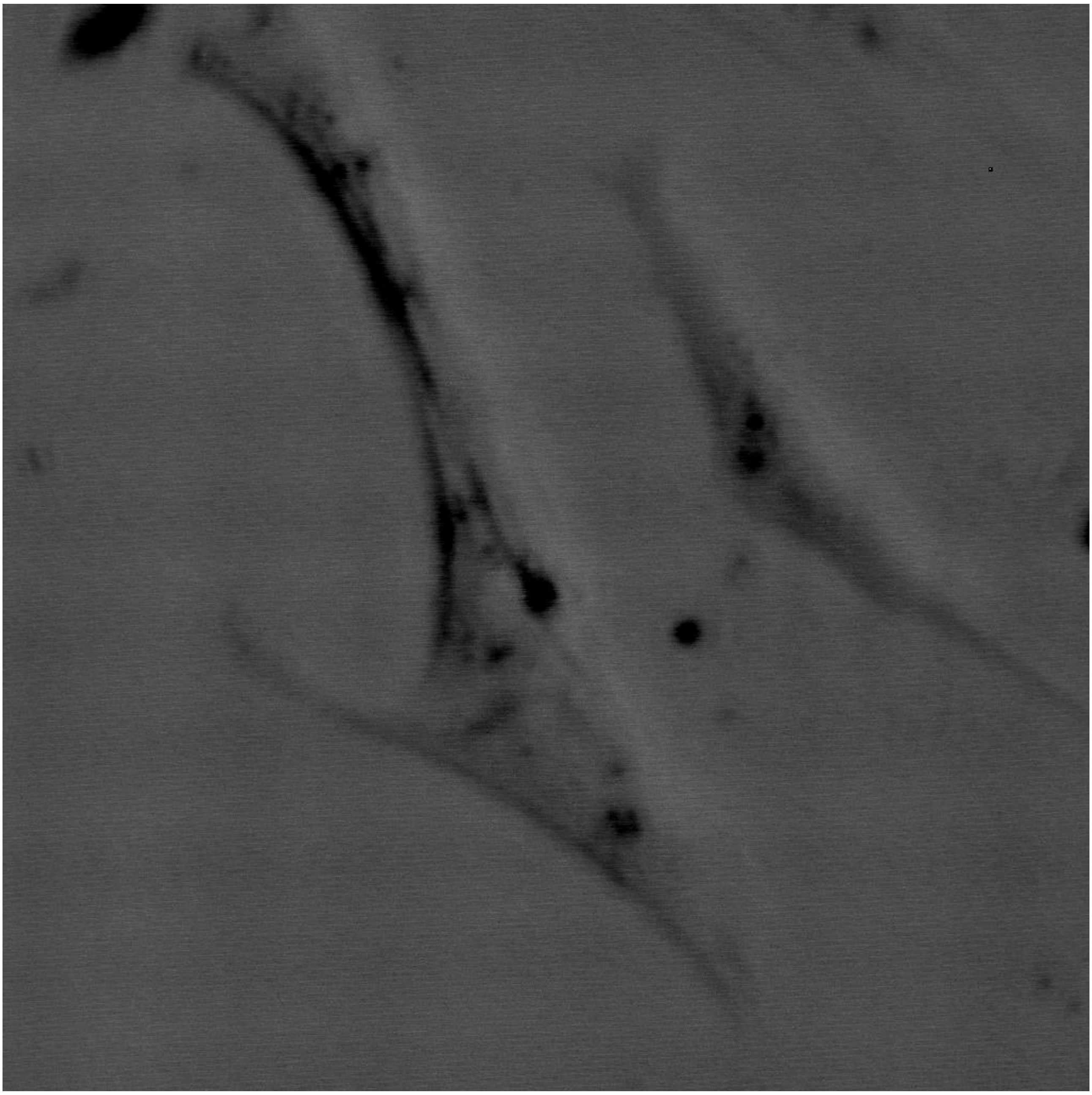

Supplementary Video S4. High-magnification confocal video of two cells showing initial localization of LNPs (green) in upper focal planes, followed by Cas9-Cy5 (red) in deeper layers, superimposed on a brightfield image. The LNPs are primarily observed near the cell periphery, while the Cas9 signal appears as punctate cytoplasmic structures, highlighting the differential intracellular distribution and potential endosomal trapping. Channels were acquired sequentially. No dye-swap or multicolor bead registration was performed; therefore, minor chromatic aberration cannot be excluded.

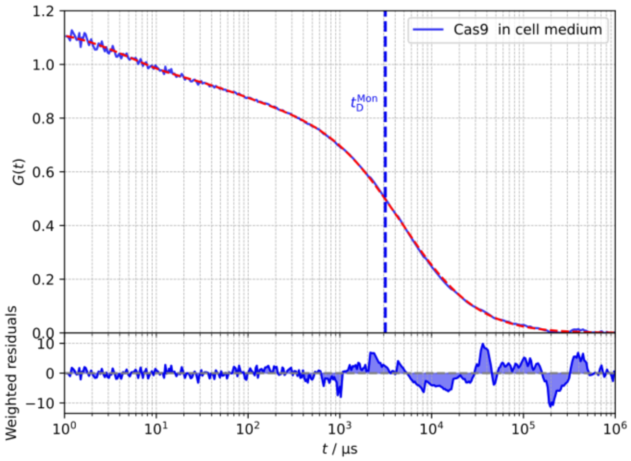

Supplementary Figure S4. Normalized fluorescence correlation curves of Cas9 labeled with Cy5 in cell medium (ThermoFisher, Opti-MEM™) (left panel) and Cas9 labeled with Cy3 in HEPES buffer, pH 7.4 (right panel). Solid lines represent experimental data, and dashed red lines indicate the model fits. Vertical dashed lines mark the fitted diffusion times t_{D} for each sample, corresponding to the monomeric $t_{\mathrm{D}}^{\mathrm{Mon}}$ and aggregated species $t_{\mathrm{D}}^{\mathrm{Agg}}$. The lower panel shows the weighted residuals of the fits.

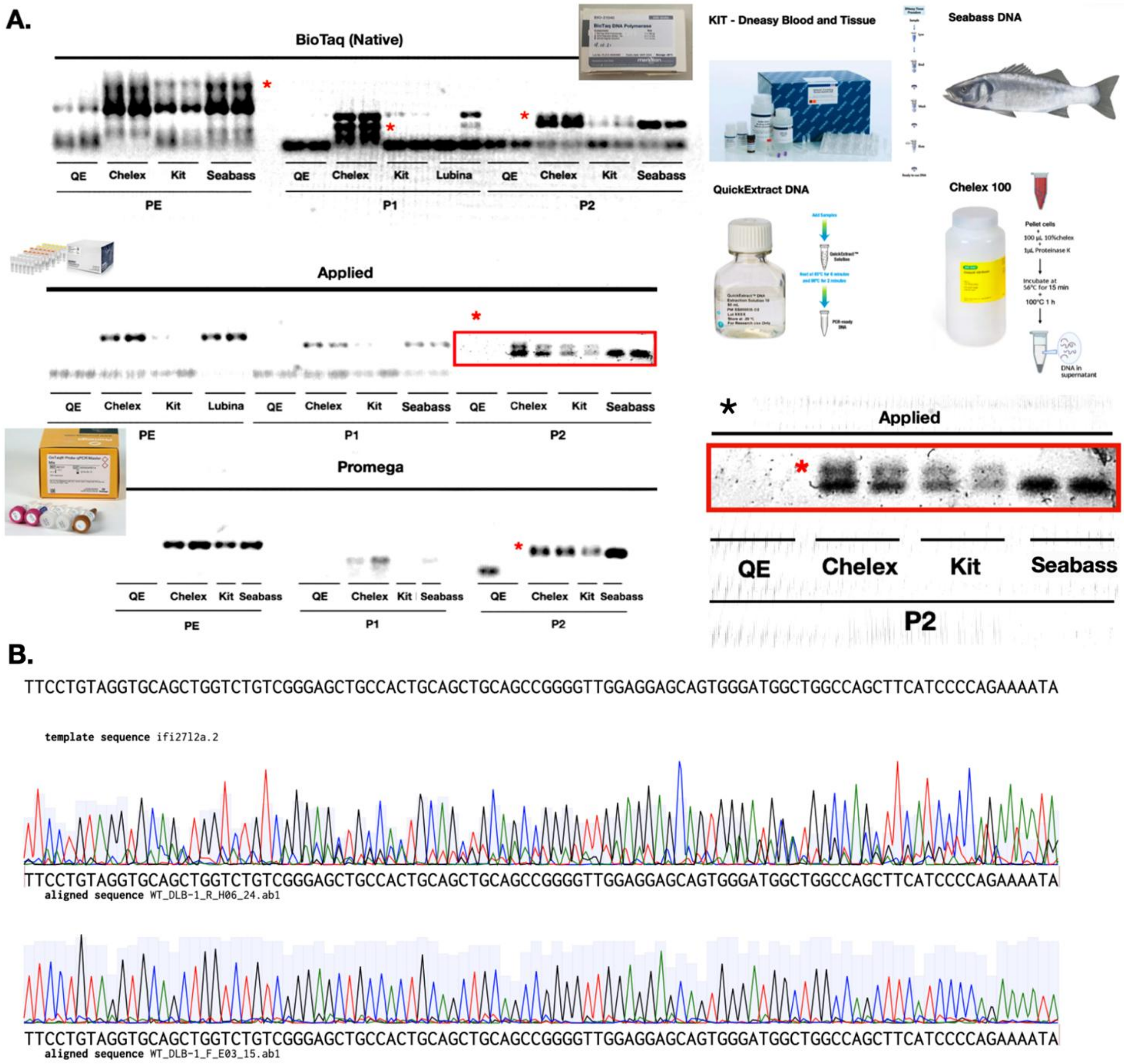

Supplementary Figure S5. PCR amplification of three genomic regions (P1, P2, PE) from SaB-1 and DLB-1 cell lines using three commercial DNA polymerases (BioTaq, Applied Biosystems, Promega) and four DNA sources: QuickExtract (QE), Chelex 100, DNeasy Blood & Tissue Kit (Qiagen), and crude seabass DNA (positive control). A. Representative 1% agarose gels showing amplification results for each enzyme–template combination. Red asterisks mark relevant anomalies: (i) overloaded wells in BioTaq reactions, and (ii) the appearance of extra PCR products, observed exclusively in cell line samples. Fragment multiplicity was consistently associated with the P2 primer pair, regardless of the polymerase used, but only when the template was derived from cultured cells (QE, Chelex, Kit). In contrast, seabass DNA yielded a single, specific amplicon for all primer sets. Additional unspecific bands were particularly prominent with the BioTaq polymerase. A black asterisk indicates the gel region shown enlarged below, highlighting that all cell line-derived templates amplified with P2 primers produced multiple bands, whereas seabass DNA generated a single product. This contrast suggests structural variation or genomic heterogeneity in the cell lines. B. Sanger sequencing chromatograms of the P2 amplicon from seabass genomic DNA amplified with ifi27l2a-2F and ifi27l2a-2R. The top trace (forward primer) shows minor baseline noise at the 5′ end; the bottom trace (reverse primer) shows a clean, unambiguous profile, confirming the presence of a single genomic target in the original species. Chromatograms were exported from Benchling (https://benchling.com).

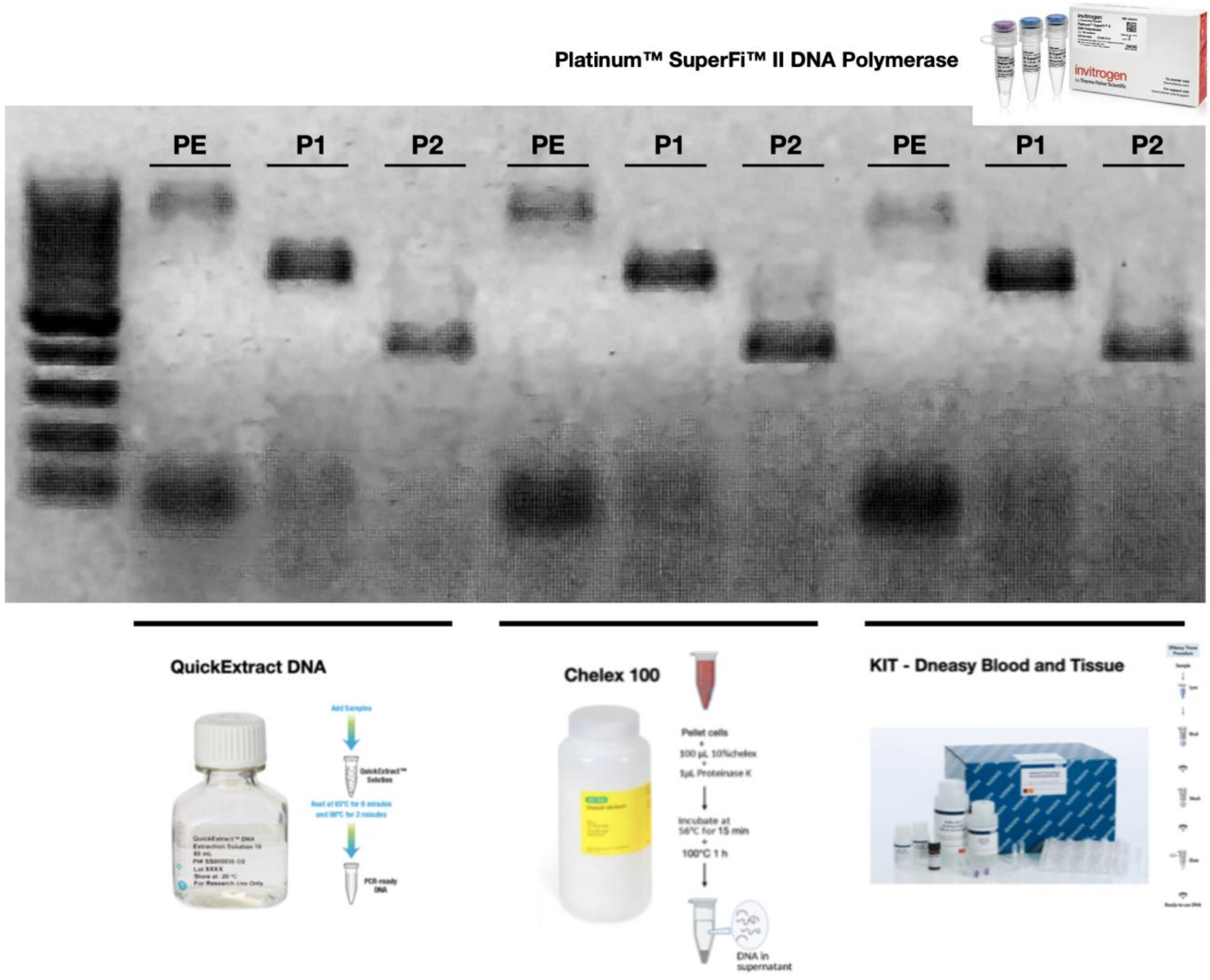

Supplementary Figure S6. Amplification of genomic DNA extracted from fish cell lines using three different methods: QuickExtract DNA, Chelex 100, and the DNeasy Blood and Tissue Kit. PCR was performed using Platinum™ SuperFi™ II DNA Polymerase, resulting in successful amplification across all extraction methods. The gel electrophoresis (1% agarose) shows consistent amplification products from each protocol, with variations in band intensity likely reflecting differences in DNA purity or yield. Representative schematics of the extraction procedures are included below each corresponding lane.

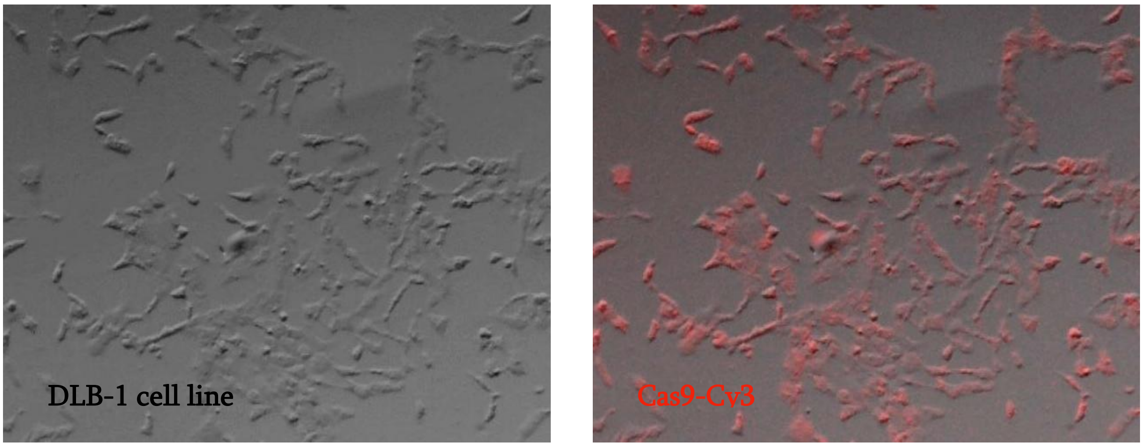

Supplementary Figure S7. Representative fluorescence microscopy image of DLB-1 cells transfected with Cas9-Cy3 RNPs using Lipofectamine 3000. Red signal (Cy3) indicates Cas9 distribution. A diffuse, non-punctate cytoplasmic signal is observed, with no evidence of nuclear accumulation or compartmental localization, consistent with inefficient intracellular trafficking and absence of genome editing.

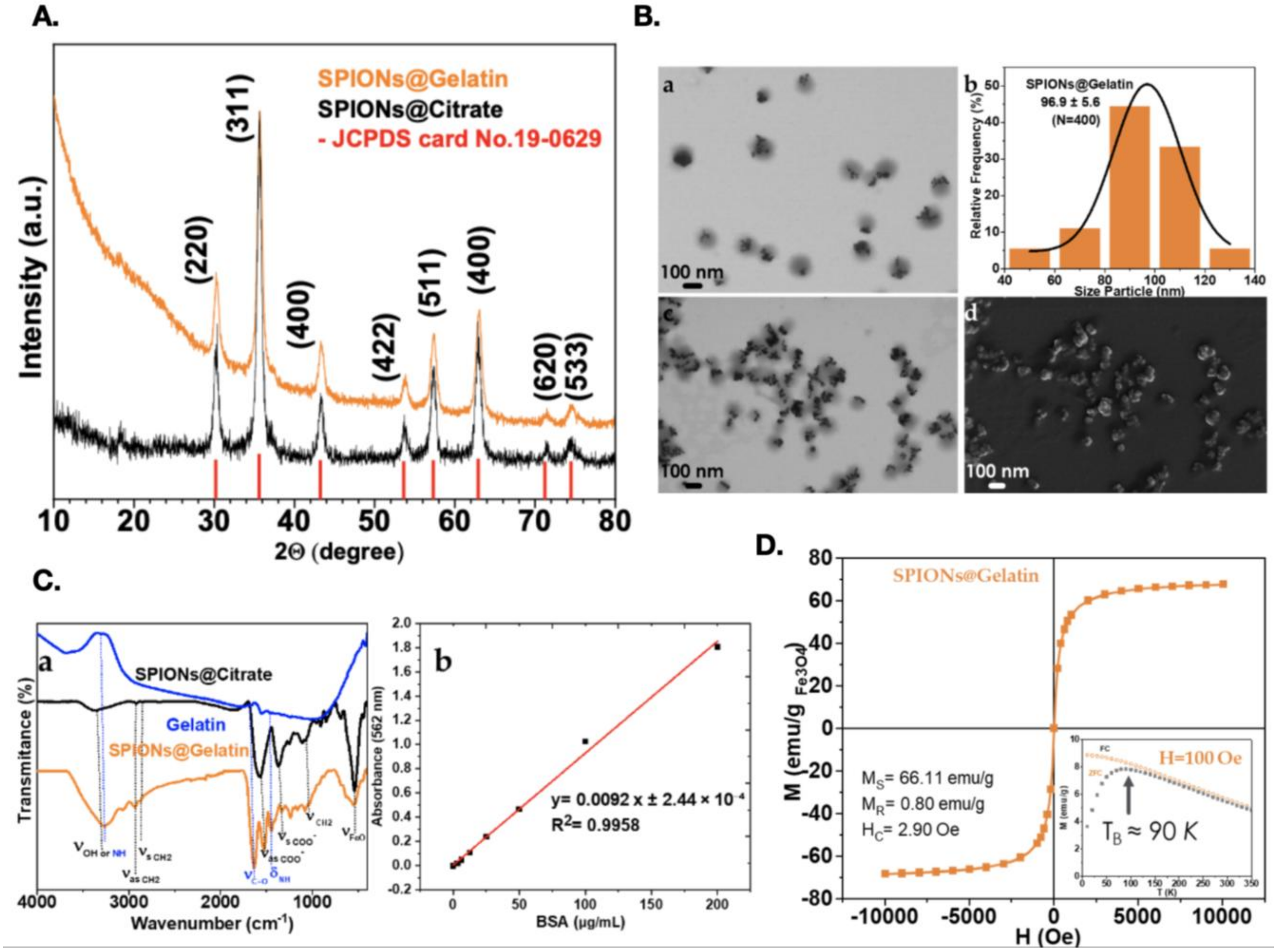

Supplementary Figure S8. A. X-ray diffraction (XRD) pattern of the SPION (blue pattern) and silica-coated SPION (black pattern) compared to the XRD pattern of magnetite from the JCPDS 19-0629 data base. B. (a) STEM micrograph of SPIONs@Gelatin showing spherical core–shell morphology with good dispersion. (b) Histogram of particle size distribution obtained from the analysis of 400 nanoparticles using ImageJ software, indicating an average diameter of approximately 100 nm. (c) Bright-field and (d) dark-field STEM images of SPIONs@Gelatin after bioconjugation with the Cas9–*sgRNA* complex, showing preservation of spherical morphology and a tendency toward aggregation. C. (a) FT-IR spectra of pure gelatin, SPIONs@Citrate and SPIONs@Gelatin and (b) Calibration curve obtained using the Micro BCA Protein Assay Kit for the quantification of Cas9–*sgRNA* complexes bioconjugated to SPIONs@Gelatin. BSA was used as the protein standard, with concentrations ranging from 0 to 200 µg/mL. Absorbance values were measured in triplicate for each concentration, and the stand-ard curve was generated by linear regression to determine protein concentrations in the samples. D. Room-temperature magnetization curves of SPIONs@Gelatin obtained using a VSM within a magnetic field range of –10 to +10 kOe. The figure also includes the ZFC and FC magnetization curves measured under an applied magnetic field of 100 Oe.
